# LIF is essential for ISC function and protects against radiation-induced gastrointestinal syndrome

**DOI:** 10.1038/s41419-020-02790-6

**Published:** 2020-07-27

**Authors:** Huaying Wang, Jianming Wang, Yuhan Zhao, Xiao Zhang, Juan Liu, Cen Zhang, Bruce Haffty, Michael Verzi, Lanjing Zhang, Nan Gao, Zhaohui Feng, Wenwei Hu

**Affiliations:** 1 Rutgers Cancer Institute of New Jersey, Rutgers University, New Brunswick, 08903 NJ USA; 2 Department of Biological Sciences, Rutgers University, Newark, 07102 NJ USA; 3 Department of Genetics, Human Genetics Institute of New Jersey, Rutgers University, Piscataway, 08854 NJ USA; 4 Department of Pathology, Penn Medicine Princeton Medical Center, Plainsboro, 08536 NJ USA

**Keywords:** Intestinal stem cells, Regeneration, Gastrointestinal diseases

## Abstract

Leukemia inhibitory factor (LIF) is a cytokine essential for maintaining pluripotency of mouse embryonic stem cells. However, its role in adult intestinal stem cells (ISCs) is unclear. The adult intestinal epithelium has a high self-renewal rate driven by ISCs in crypts. Here, we find that LIF is present in the ISC niche in crypts and critical for the function of ISCs in maintaining the intestinal epithelial homeostasis and regeneration. Mechanistically, LIF maintains β-catenin activity through the AKT/GSK3β signaling to regulate ISC functions. LIF deficiency in mice impairs the renewal of the intestinal epithelium under the physiological condition. Further, LIF deficiency in mice impairs the regeneration of intestinal epithelium in response to radiation and shortens the lifespan of mice after high doses of radiation due to gastrointestinal (GI) syndrome, which can be rescued by administering recombinant LIF (rLIF). Importantly, LIF exhibits a radioprotective role in wild-type (WT) mice by protecting mice from lethal radiation-induced GI syndrome; administering rLIF promotes intestinal epithelial regeneration and prolongs survival in WT mice after radiation. These results reveal a previously unidentified and a crucial role of LIF in ensuring ISC function, promoting regeneration of the intestinal epithelium in response to radiation and protecting against radiation-induced GI syndrome.

## Introduction

LIF is a multi-functional cytokine and plays an important role in various biological processes^1,2^. Previously, we found that LIF is a target gene of tumor suppressor p53, and mediates p53’s function in regulation of embryonic implantation in mice and humans^3,4^. To exert its function, LIF binds to its receptor complex composed of LIF receptor and glycoprotein gp130 to activate several signaling pathways, including the JAK/STAT, MAPK, and PI3K/AKT pathways, in a highly cell- and tissue-specific manner^1,2,5^. LIF is crucial in maintaining the pluripotency of mouse embryonic stem cells^6^. However, basal LIF expression levels are generally low in normal adult tissues and the role of LIF in adult stem cells in somatic tissues is not well-understood.

The intestinal crypt drives rapid self-renewal of the intestinal epithelium, which is one of the best-defined adult stem cell models^7^. The intestinal crypt contains Lgr5^+^ intestinal stem cells (ISCs), transit-amplifying (TA) cells, and Paneth cells. Under physiological conditions, ISCs generate precursors of enterocytes and secretory cells that divide and differentiate into enterocytes, goblet, enteroendocrine and tuft cells. Upon injury, such as ionizing radiation (IR), intestinal epithelial progenitor cells and mature epithelial cells have a remarkable plasticity in their capabilities to contribute to epithelial regeneration^8–10^. A number of signaling pathways, including the β-catenin signaling, are involved in the regulation of ISC function within the ISC niche^11,12^.

In this study, we revealed a previously unidentified and essential role of LIF in maintaining ISC function in vivo. LIF is expressed in the ISC niche in the intestinal tissues in both mice and humans. LIF maintains ISCs’ function by up-regulating β-catenin activity mainly through the AKT signaling. LIF deficiency in mice impairs the homeostasis of intestinal epithelium under the physiological condition and the regeneration of intestinal epithelium after injury in response to radiation. Administering recombinant LIF (rLIF) protein rescues the impairment of the regeneration of intestinal epithelium after injury induced by radiation in LIF deficient mice. Further, rLIF shows a radioprotective role in wild-type (WT) mice through promoting the regeneration of intestinal epithelium after radiation and in turn protecting mice from radiation-induced gastrointestinal (GI) syndrome. Results from this study reveal a crucial role of LIF in supporting ISC function, promoting regeneration of intestinal epithelium in response to radiation, and protecting against radiation-induced GI syndrome.

## Materials and methods

### Mouse strains and IR treatment

Conventional LIF KO mice established by Dr. Stewart^13^ in C57BL/6J background were obtained from EMMA repository (EM:02619). Lgr5-EGFP-IRES-creERT2 mice (Lgr5-GFP) were obtained from the Jackson Laboratory. Age- and gender-matched LIF KO and WT mice at 8–12-week-old were used. Animals were randomly assigned to different treatment groups. For survival experiment, at least 5 mice/group were used and at least 3 mice/group were used for other experiments. Sample sizes were chosen based on the power calculation. For ionizing radiation (IR) treatment, mice were subjected to 9 or 12 Gy whole-body IR with a 137 Cs γ-source irradiator at a dose rate of 90 cGy/minute. For LIF treatment, mice were randomly assigned to groups with intraperitoneal (*i.p*.) injection of either mouse rLIF (Millipore, 30 ng/g body weight) or vehicle control twice a day for 7 days. The investigators were blinded to the group allocation during the experiment and when assessing the outcome. All animal experiments were approved by the Institutional Animal Care and Use Committee (IACUC) of Rutgers University.

### Crypt isolation and organoid culture

Crypt isolation and organoid culture were performed as previously described^14^. Serial organoid passaging was performed every six days as previously described^15^. Mouse rLIF (50 ng/ml, Millipore), Wortmannin (1 µM, Cell Signaling), Capivasertib (1 µM, MCE), SC79 (5 µM, Sigma), Stattic (2 µM, Sigma), SB242235 (1 µM, MCE) and CHIR99021 (3 µM, Stemgent) were used for organoid treatments. The size of organoids was evaluated by quantifying the surface area of horizontal cross section of organoids acquired from multiple random non-overlapping pictures using the ImageJ software.

### Flow cytometry assays

Flow cytometry assays were used to measure Lgr5-GFP positive cells in the small intestine and colon of Lgr5-GFP mice as previously described^16^. Propidium iodide (Millipore) was used to exclude dead cells.

### Histology

Paraffin-embedded small intestine tissues were stained with hematoxylin and eosin (H&E) as previously described^17^. Briefly, after flushing with ice-cold PBS, intestinal tissues were coiled into “Swiss rolls” and fixed in 10% formalin solution for 48 h. Tissues were then embedded in paraffin and sectioned at a thickness of 5 µm. The villus length and density, and the crypt depth were quantified by using the ImageJ software.

### IHC staining assays

De-identified normal human colorectal tissue samples were obtained from Princeton Cancer Tissue Repository with an IRB approval. Patient consents were obtained at the time of tissue collection. IHC staining was performed as previously described^14^. Briefly, tissue sections were deparaffinized in xylene and rehydrated in ethanol and water, followed by antigen retrieval by boiling slides in antigen unmasking solution (Vector Laboratories) for 10 min. Immunostaining was performed using the following antibodies: anti-LIF (Novus, 1:500), anti-lysozyme (Abcam, 1:5000), anti-Olfm4 (Cell signaling, 1:1000), anti-β-catenin (BD, 1:500), anti-CD44 (BioLegend, 1:500), anti-Ki67 (Abcam, 1:200), anti-DCLK (Abcam, 1:200), and anti-cleaved caspase 3 (Cell signaling, 1:500) antibodies.

### Alcian blue staining assays

Paraffin-embedded small intestine tissues were stained with alcian blue to detect goblet cells as previously described^18^.

### IF staining assays

IF staining of organoids was performed as previously described^19^. Anti-Ki67 (Abcam, 1:250), anti-GFP (Abcam, 1:1000) or FITC-anti-UEA-1 (Sigma, 1:1000) antibodies were used for staining. For IF staining of mouse intestine tissues, tissue sections were deparaffinized in xylene and rehydrated with ethanol. After pre-incubation with 2% BSA and 2% goat serum, tissue sections were incubated with the anti-LIF (Novus, 1:500), anti-lysozyme (Abcam, 1:5000), anti-Olfm4 (Cell signaling, 1:1000) or anti-GFP (Abcam, 1:1000) antibodies overnight at 4° C. Slides were then incubated with Alexa Fluor® 555 Goat Anti-Rabbit IgG (H + L) or Alexa Fluor® 488 Goat Anti-mouse IgG (H + L) (1:200). Nuclei were stained with 4’, 6-diamidino-2-phenylindole (DAPI; Vector labs).

### Western blot assays

Standard Western blot assays were used to analyze protein expression in small intestinal tissues. Briefly, tissues were lysed using the lysis buffer (10Mm Tris, 100 mM NaCl, 1 mM EDTA, 1 mM EGTA, 1 mM NaF, 20 mM Na_4_P_2_O_4_, 2 mM Na_3_VO_4_, 1% Triton X-100, 10% Glycerol, 0.1% SDS, 0.5% Deoxycholate Acid). Protein lysates were separated on a 4 to 20% Tris-glycine gel and transferred to a polyvinylidene difluoride membrane. The following antibodies were used: anti-AKT (Santa Cruz, 1:2000), anti-p-AKT-Ser473 (Cell Signaling, 1:2000), anti-GSK3β (Cell signaling, 1:1000), anti-p-GSK3β-Ser9 (Abcam, 1:2000) and anti-β-actin (Sigma, 1:100000) antibodies.

### Quantitative real-time PCR assays

Quantitative real-time PCR assays were performed as previously described^17^. The sequences of primers used for real-time PCR assays were listed in Supplementary Table S1. The mRNA expression of genes was normalized with the β-actin gene.

### Statistical analysis

All data were obtained from at least three repetitions and were expressed as mean ± SD. The Kolmogorov–Smirnov test was used to analyze data distribution. Data with normal distribution and homogeneity of variance were used for further analysis. The difference between two groups in experiments containing multiple groups were analyzed for statistical significance by One-way analysis of variance (ANOVA) followed by Student–Newman–Keuls (SNK) test. Fisher’s Exact Test were used to analyze the difference of the percentage of organoid formation between groups. The survival of mice after IR was summarized by Kaplan–Meier plots and compared using the log-rank test using GraphPad Prism software. All other *p*-values were obtained using Student’s *t-*tests. Values of *p* < 0.05 were considered to be significant.

## Results

### LIF is expressed in intestinal crypts

**Fig. 1:**
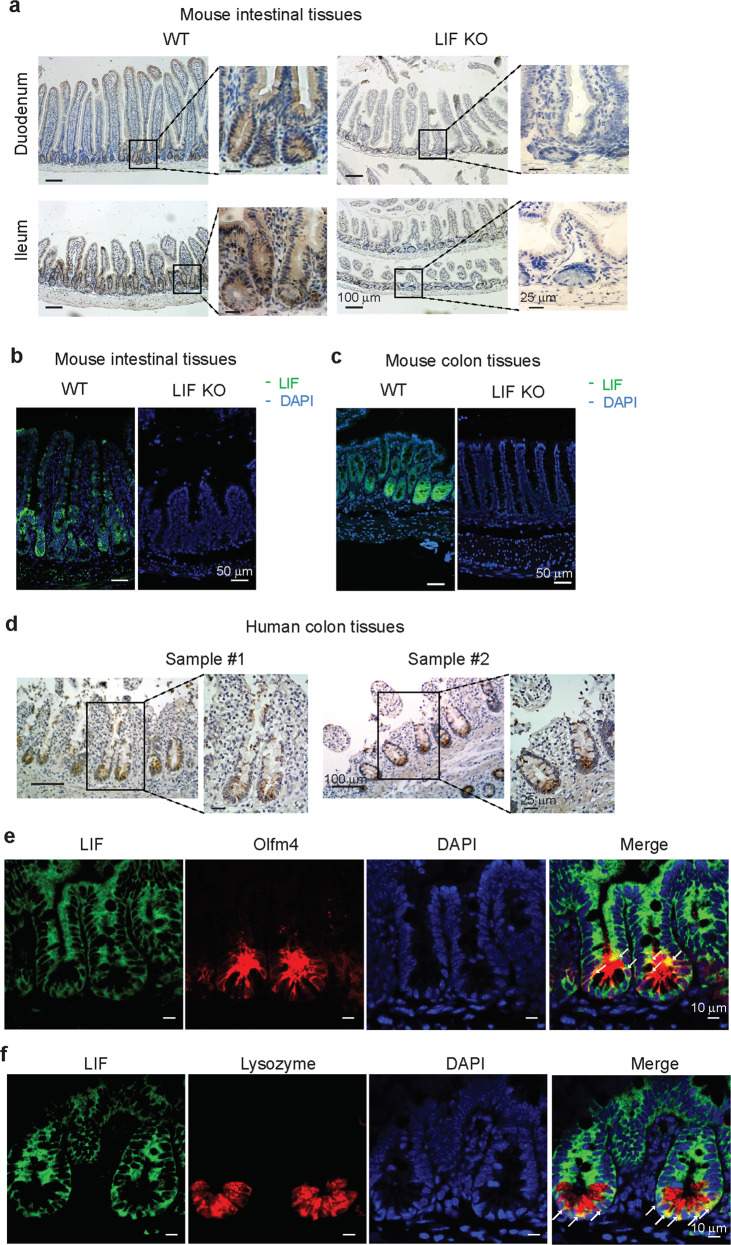
The expression of LIF in the intestinal epithelium. **a**, **b** LIF expression in the intestinal crypts was determined by **a** IHC staining of the duodenum and ileum, and **b** IF staining of the small intestine of WT and LIF KO mice. **c** LIF expression in the mouse colon was determined by IF staining. **d** LIF expression in human colon tissues was determined by IHC staining. **e**, **f** Co-localization of LIF with Olfm4 (**e**) and Lysozyme (**f**) was observed in the small intestine of WT mice as determined by IF staining. The white arrow represents the co-localization of LIF with Olfm4 (**e**) and Lysozyme (**f**), respectively.

We investigated the expression pattern of LIF in the mouse intestinal tissue by immunohistochemistry (IHC) and immunofluorescent (IF) staining using a LIF antibody. The mouse intestinal tissue from LIF knockout (KO) mice was used to ensure antibody specificity. LIF expression was observed in the epithelia along the entire intestine including duodenum, ileum and colon in WT mice (Fig. 1a–c). Majority of the cells with positive LIF staining were localized in the crypt in WT mice (Fig. 1a–c). LIF was expressed at very low levels in the intestinal epithelium in the embryos at E14.5 and E18.5 (Supplementary Fig. S1). The expression of LIF in the intestinal epithelium was readily observed after birth as determined at P7 and P15, with majority of LIF positive cells localized in the crypt (Supplementary Fig. S1). LIF expression was also observed in the epithelial cells in the crypt of normal human colon tissues (Fig. 1d).

The intestinal crypts contain different types of cells, including ISCs, Paneth cells and TA cells. Using Olfm4 and Lysozyme, markers of ISCs and Paneth cells, respectively, for co-staining, we found that LIF was expressed in subsets of ISCs and Paneth cells, as well as some TA cells (Fig. 1e, f).

These results demonstrate that LIF is expressed in intestinal crypt epithelial cells.

### LIF deficiency impairs development of the small intestine in mice

**Fig. 2:**
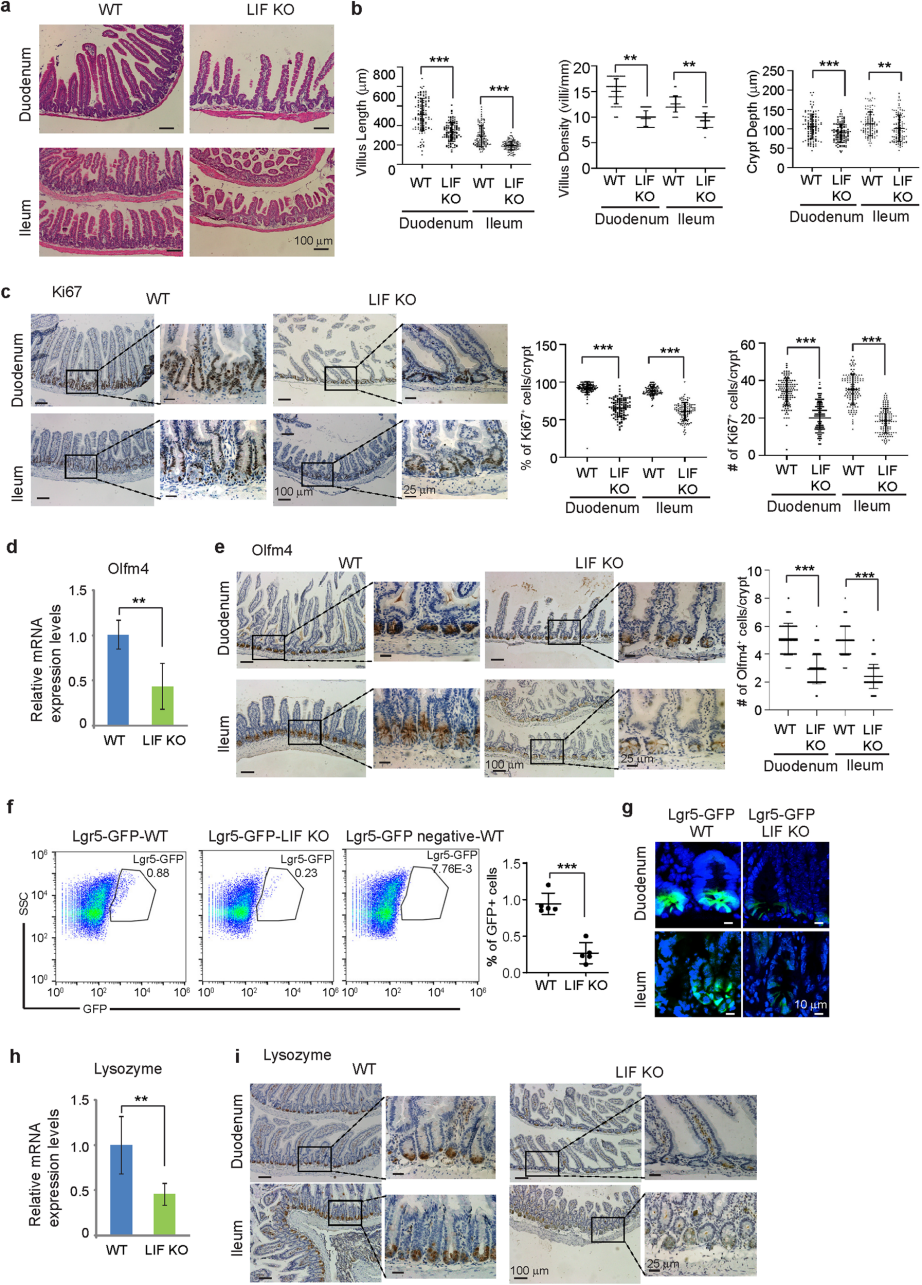
LIF deficiency impairs the development of the small intestine in mice. **a** Representative H&E staining images of the duodenum and ileum of WT and LIF KO mice. **b** Quantification of the villus length (*n* = 120 villi from at least 3 mice/group), villus density (*n* = 48 fields from at least 3 mice/group), and crypt depth (*n* = 120 crypts from at least 3 mice/group) in the small intestine of WT and LIF KO mice. The villus length and crypt depth were normalized to the average weight of WT mice with the same age and gender. **c** A significantly decreased number and percentage of proliferating cells in the crypts of LIF KO mice. Left panels: representative images of IHC staining of Ki67. Right panels: quantification of the percentage and number of Ki67 positive cells/crypt. *n* = 120 crypts from at least 3 mice/group. **d**–**g** The number of ISCs was greatly reduced in LIF KO mice compared with WT mice. **d** The mRNA level of Olfm4 in the small intestine of mice was determined by real-time PCR assays and normalized with β-actin. *n* = 6 mice/group. **e** IHC staining of Olfm4 in the duodenum and ileum of mice. Left panels: representative IHC images. Right panel: quantification of the number of Olfm4^+^ cells/crypt. *n* = 120 crypts from at least 3 mice/group. **f** The percentage of Lgr5-GFP^+^ cells in small intestinal epithelial cells was decreased in LIF KO; Lgr5-GFP mice compared with Lgr5-GFP^+^ WT mice. Left panels: representative images of flow cytometry analysis. Right panel: quantification of the percentage of Lgr5-GFP^+^ cells in small intestinal epithelial cells. *n* = 5 mice/group. **g** IF staining of GFP in the duodenum and ileum of WT; Lgr5-GFP and LIF KO; Lgr5-GFP mice. **h**, **i** The number of Paneth cells was greatly reduced in LIF KO mice. **h** The mRNA level of Lysozyme in the small intestine of mice. *n* = 6/group. **i** IHC staining of Lysozyme in the duodenum and ileum of mice. In **b**–**f** & **h**, data are presented as mean ± SD. **p < 0.01, ***p < 0.001; Student’s *t*-test.

While LIF is essential for the maintenance of pluripotency of mouse embryonic stem cells, its role in regulation of adult ISCs is unclear. Breeding of LIF heterozygous mice to generate LIF KO mice showed that while E14.5-day embryos exhibited a Mendelian distribution of LIF KO allele, offspring mice at the weaning-age exhibited a non-Mendelian inheritance of the LIF KO allele; homozygous LIF KO offspring mice were fewer than the predicted ratios (Supplementary Fig. S2a). Viable LIF KO mice showed a retarded postnatal growth rate which resulted in ~20% lower body weights compared with WT mice (Supplementary Fig. S2b), which is consistent with a previous study^13^. Histological analysis revealed that the length and the density of intestinal villi in LIF KO mice were significantly reduced compared with WT mice (Fig. 2a, b), although different differentiated cell types, including goblet and tuft cells, were present in the intestinal villi in LIF KO mice (Supplementary Fig. S3a, b). In addition, a marked decrease in the crypt size along the entire small intestine and colon was detected in LIF KO mice (Fig. 2a, b and Supplementary Fig. S3c). These results suggest that LIF is essential for intestinal epithelial homeostasis.

The homeostasis of the intestinal epithelial cells (IECs) is balanced between proliferation and apoptosis. IECs are replaced every 4–5 days through the continuous renewal, migration of new IECs and apoptosis of aged IECs. We examined intestinal epithelial proliferation by IHC staining of Ki67. In the small intestine of WT mice, Ki67 positive (Ki67^+^) cells were identified from position +4 counting from the bottom of the crypt and upwards to mark the TA population^7^. LIF deficiency led to a significant decrease in this proliferative population in the small intestine in terms of both the number and the percentage of Ki67^+^ cells per crypt (Fig. 2c). However, LIF deficiency did not have an apparent effect on apoptosis of the intestinal epithelium as determined by IHC staining of cleaved caspase-3 (Supplementary Fig. S4). These results indicate that the epithelial phenotypes observed in the LIF KO intestine are likely due to an impaired epithelial proliferation rather than increased apoptosis.

To investigate the effect of LIF deficiency on ISCs, the expression of ISC marker Olfm4 was examined at both RNA and protein levels by real-time PCR and IHC staining assays, respectively. Compared with WT mice, Olfm4 mRNA and protein levels, and Olfm4^+^ cells per crypt were dramatically reduced in the crypt of LIF KO mice (Fig. 2d, e). The Lgr5-EGFP-IRES-creERT2 mice (namely Lgr5-GFP mice) with a knock-in allele expressing GFP from the Lgr5 locus are widely used for the identification of ISC population^20^. We used Lgr5-GFP mice to re-examine ISCs in the crypt in LIF KO mice. LIF KO; Lgr5-GFP mice displayed a reduced number of Lgr5-GFP positive cells and reduced GFP protein levels in remaining positive cells in crypts from the small intestine as determined by flow cytometry and IF staining assays, respectively (Fig. 2f, g). Similarly, LIF KO; Lgr5-GFP mice displayed a reduced number of Lgr5-GFP positive cells in crypts from the colon (Supplementary Fig. S5a). Furthermore, the levels of CD44, another ISC marker^21^, were greatly reduced in the crypt of LIF KO mice compared with WT mice (Supplementary Fig. S5b).

Paneth cells constitute the niche for stem cells in the intestinal crypt and are the major supportive epithelial cells for ISCs^14^. Compared with WT mice, the levels of lysozyme, a Paneth cell marker, were clearly reduced in the intestinal tissue of LIF KO mice at both mRNA and protein levels (Fig. 2h, i). Furthermore, LIF KO mice displayed an overall decreased fluorescence intensity of lysozyme staining and a significantly reduced Paneth cell count per crypt (Supplementary Fig. S6a–c).

Collectively, our results show that LIF deficiency leads to the decreased number of ISCs and Paneth cells in crypts and reduced proliferation of intestinal epithelium cells, which impairs the homeostasis of self-renewing small intestinal crypts.

### LIF deficiency impairs growth of the intestinal organoids

**Fig. 3:**
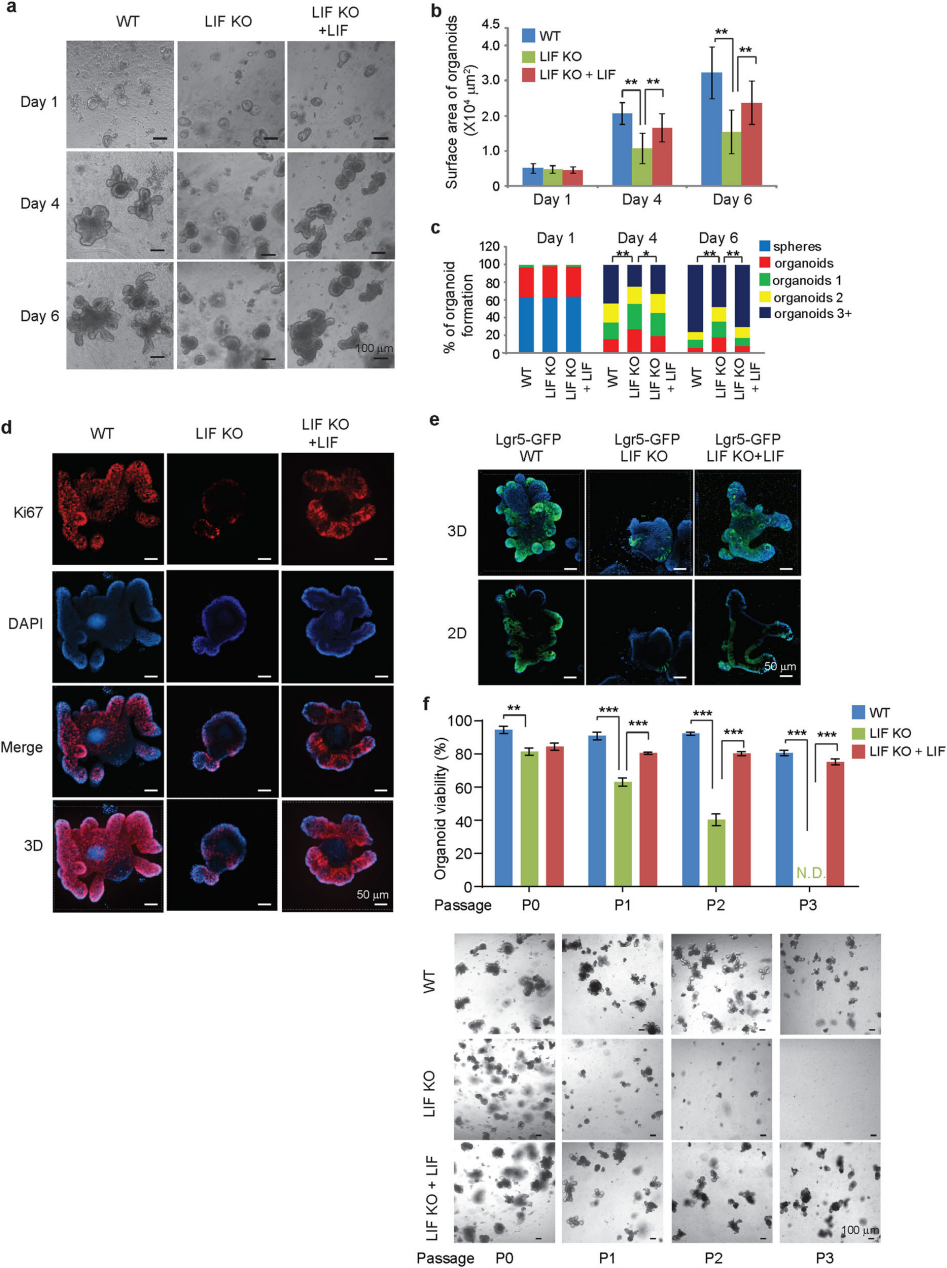
LIF deficiency impairs the growth of intestinal organoids. **a** Representative images showing the growth of intestinal organoids from WT and LIF KO mice. For LIF treatment, LIF (50 ng/mL) was added into the medium. **b** Quantification of organoid surface area. Data are presented as mean ± SD. *n* ≥ 30/group, **p < 0.01; One-way ANOVA followed by SNK test. **c** Quantification of the percentage of organoid formation. Organoids, organoids 1, 2, and 3+ refers to organoids with no bud, one bud, two buds and three or more buds, respectively. *n* ≥ 100/group, *p < 0.05; **p < 0.01; Fisher’s exact test. **d** IF staining of Ki67 in the intestinal organoids. **e** IF staining of GFP in the intestinal organoids from WT; Lgr5-GFP and LIF KO; Lgr5-GFP mice. **f** Intestinal organoids derived from LIF KO mice showed significantly reduced ability to regenerate complete organoids upon passaging (P). Top panel: quantification of the percentage of budded organoids after passaging. Data are presented as mean ± SD. *n* = 4/group. **p < 0.01; ***p < 0.001; Student’s *t*-test. Bottom panels: representative images showing the growth of organoids 3 days after each passaging.

Next, we examined whether LIF regulates the function of ISC compartment by employing an organoid model of ex vivo epithelial regeneration^14^. Compared with WT crypts, LIF KO crypts exhibited a significantly reduced ability to proliferate, expand and form budding organoids (Fig. 3a–c). The growth of LIF KO organoids was much slower than WT organoids, with significantly reduced epithelial buds (Fig. 3a–c). Notably, the impaired growth of LIF KO organoids was largely rescued by supplementing culture media with mouse rLIF (Fig. 3a–c). Similarly, the number of Ki67^+^ cells was greatly decreased in LIF KO organoids, which was largely restored by the supplementation of rLIF (Fig. 3d). The number of Paneth cells as determined by IF staining of UEA-1, a Paneth cell marker^22^, was also decreased in LIF KO organoids, which was largely restored by the supplementation of rLIF (Supplementary Fig. S6d). Organoids formed from LIF KO; Lgr5-GFP mouse crypts displayed dramatically decreased Lgr5-GFP levels compared with WT organoids as determined by IF staining, and the reduction was largely rescued by the supplementation of rLIF (Fig. 3e). Unlike WT organoids, which grow indefinitely, LIF KO organoids had a lower survival rate after passaging, and died out after 3 passages, indicating that LIF KO organoids had a reduced self-renewal capacity compared with WT organoids (Fig. 3f). The impaired self-renewal ability of LIF KO organoids was largely rescued by supplementing with rLIF (Fig. 3f). Consistently, administering mouse rLIF (i.p., 30 ng/g body weight, twice a day for 7 days) greatly rescued the impaired intestinal homeostasis in LIF KO mice. LIF KO mice administered with rLIF exhibited a significantly increased length and density of intestinal villi, the depth of crypt, the number and percentage of Ki67^+^ cells per crypt and the number of Olfm4^+^ cells per crypt (Supplementary Fig. S7). These results demonstrate an important role of LIF in maintaining the clonogenic activity of ISCs.

### LIF deficiency inhibits the β-catenin signaling in the small intestine

**Fig. 4:**
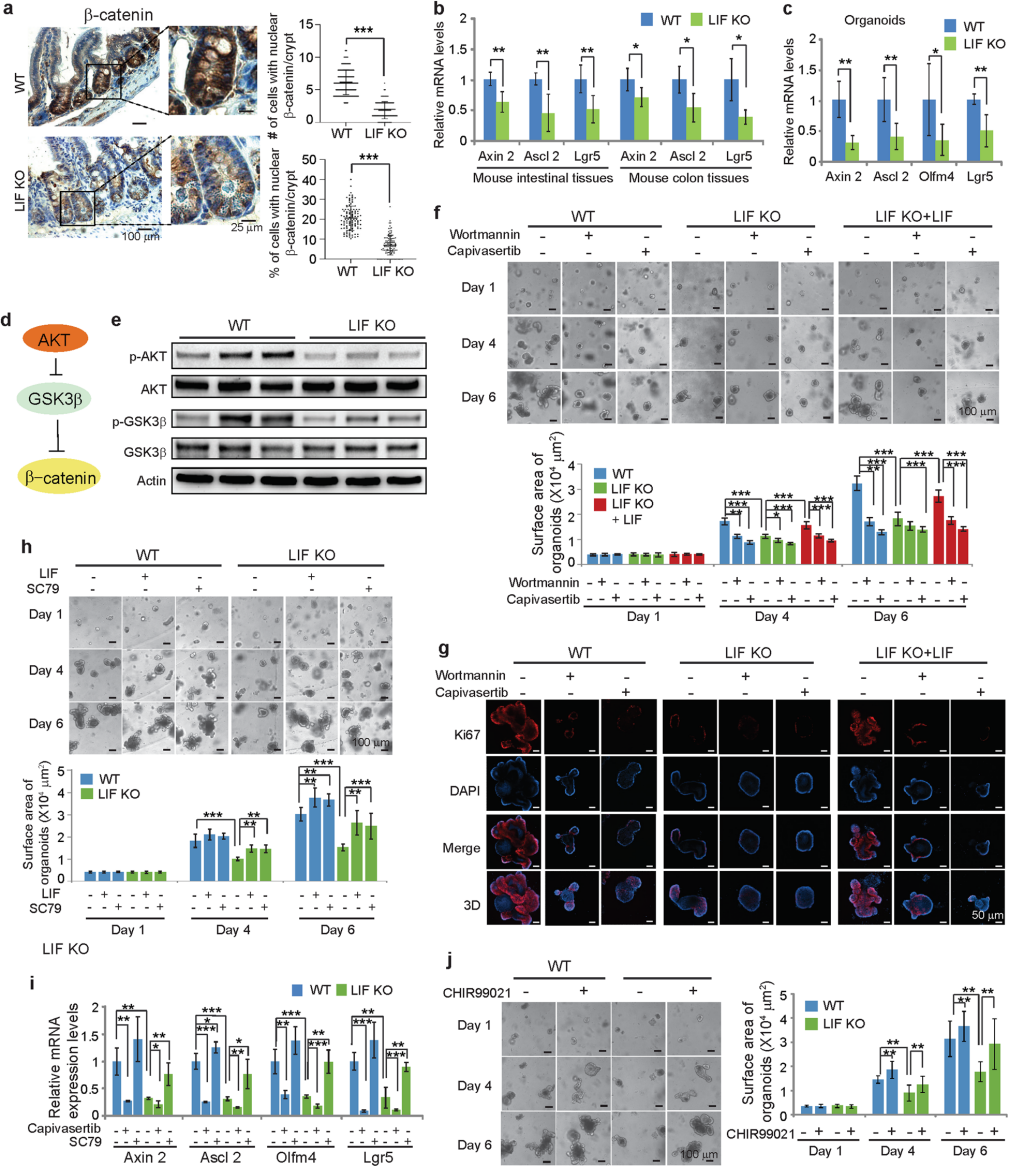
LIF deficiency inhibits the β-catenin signaling pathway *via* the AKT singaling in the small intestine of mice. **a** The mouse small intestine of LIF KO mice had significantly less nuclear β-catenin staining compared with WT mice. Left panels: IHC staining of β-catenin in the small intestine. Right panels: Quantification of the number (top) and percentage (bottom) of cells with positive nuclear β-catenin staining/crypt. *n* = 120 crypts from at least 3 mice/group. **b**, **c** Relative mRNA expression levels of a group of well-known β-catenin target genes in the small intestine and colon (**b**) and organoids (**c**) from WT and LIF KO mice. *n* = 6 mice/group. **d** A schematic model depicting the regulation of β-catenin through AKT. **e** Decreased phosphorylation levels of AKT Ser-473 (p-AKT) and GSK3β Ser-9 (p-GSK3β) in the small intestine of LIF KO mice as determined by Western-blot assays. **f**, **g** The PI3K/AKT inhibitor Wortmannin and AKT inhibitor Capivasertib inhibited the growth and proliferation of WT organoids and LIF KO organoids supplemented with rLIF, but had a much less pronounced effect on LIF KO organoids. **f** Top panels: representative images showing the growth of organoids with or without Wortmanin (1 µM) or Capivasertib (1 µM) treatment. Bottom panel: quantifications of organoid surface area. **g** IF staining of Ki67 in WT, LIF KO and LIF KO + LIF intestinal organoids with or without Wortmanin or Capivasertib treatment at day 6. **h** The AKT agonist SC79 significantly enhanced the growth of LIF KO organoids. Top panels: representative images showing the growth of organoids with or without LIF (50 ng/ml) or SC79 (5 µM) treatment. Bottom panel: quantifications of the surface area of organoids. **i** Relative mRNA expression levels of Axin2, Ascl2, Olfm4 and Lgr5 in WT and LIF KO intestinal organoids with or without Capivasertib or SC79 treatment. *n* = 4 mice/group. **j** The GSK3β inhibitor CHIR99021 rescued the impaired growth of LIF KO organoids. Left panels: representative images showing the growth of organoids with or without CHIR99021 treatment (3 µM). Right panel: quantifications of the surface area of organoids. In **a**–**c**, **f**, **h**–**j**, data are presented as mean ± SD. *p < 0.05; **p < 0.01; ***p < 0.001; Student’s *t*-test in **a**–**c**; One-way ANOVA followed by SNK test in **f**, **h**–**j**. For **f**, **h**, **j**, *n* ≥ 30/group.

Proper β-catenin signaling is critical for ISC function and intestinal crypt homeostasis^11,12^. The nuclear accumulation of β-catenin at the bottom of crypts in the small intestine functions as a co-activator of TCF/ LEF proteins to regulate the expression of a group of genes, and thus plays a crucial role in maintaining crypt stem/progenitor cell compartments and the homeostasis of intestinal epithelium^11,12^. IHC assays showed that LIF deficiency significantly reduced nuclear β-catenin accumulation in crypts in the small intestines (Fig. 4a). mRNA levels of a panel of well-known β-catenin/TCF target genes, including Axin2, Ascl2 and Lgr5^23^, were significantly decreased in the small intestine and colon of LIF KO mice compared with those of WT mice (Fig. 4b). Olfm4 is also a β-catenin target gene, which is expressed in the small intestine but not the colon of mice^24^. The mRNA levels of Olfm4 were significantly decreased in the small intestine of LIF KO mice (Fig. 2d). Similar reductions in mRNA levels of Axin2, Ascl2, Olfm4 and Lgr5 were observed in LIF KO organoids compared with WT organoids (Fig. 4c). These results indicate that LIF deficiency decreases the nuclear accumulation and transcriptional activity of β-catenin in crypts, which in turn impairs ISC function and homeostasis of the intestinal epithelium.

### LIF upregulates the β-catenin signaling *via* the AKT signaling in the small intestine

AKT is an important downstream target of LIF, which mediates many important functions of LIF^25,26^. Currently, it is unclear whether the LIF/AKT signaling regulates ISC function and homeostasis of the intestinal epithelium. AKT has been reported to phosphorylate GSK3β at Ser-9 to inactivate GSK3β in different types of cells, which in turn stabilizes β-catenin^27^ (Fig. 4d). We found that LIF deficiency decreased the AKT activity in the small intestine as reflected by the decreased levels of AKT phosphorylation at Ser-473 (p-AKT) in the small intestine of LIF KO mice compared with that of WT mice (Fig. 4e). LIF deficiency decreased GSK3β Ser-9 phosphorylation levels in the small intestine (Fig. 4e), indicating that LIF deficiency leads to increased GSK3β activity to promote β-catenin degradation and inhibit its function.

To investigate whether LIF regulates ISC function through upregulating the AKT signaling, WT and LIF KO organoids were treated with Wortmannin, a PI3K/AKT inhibitor, and Capivasertib, an AKT inhibitor^28,29^. Both Wortmannin and Capivasertib greatly inhibited the growth of WT organoids and decreased cell proliferation as determined by analyzing the surface area of organoids at different days of treatment as indicated in the figure, percentage of organoid formation, and number of Ki67^+^ cells, respectively, at the end of treatment (Fig. 4f, g and Supplementary Fig. S8a). In contrast, the inhibitory effects of Wortmannin and Capivasertib on organoid growth and cell proliferation were much less pronounced in LIF KO organoids (Fig. 4f, g and Supplementary Fig. S8a). Notably, while supplementation of mouse rLIF largely rescued the impaired growth of LIF KO organoids, Wortmannin and Capivasertib treatments largely abolished the rescue effect of rLIF on the growth of LIF KO organoids (Fig. 4f, g and Supplementary Fig. S8a). SC79, an AKT agonist, greatly improved the growth and cell proliferation of LIF KO organoids but displayed a less pronounced effect on WT organoids (Fig. 4h and Supplementary Fig. S8b, c). Notably, SC79 rescued the impaired growth of LIF KO organoids to a similar extent as mouse rLIF did (Fig. 4h and Supplementary Fig. S8b, c). Capivasertib treatment significantly decreased the mRNA levels of Axin2, Ascl2, Olfm4 and Lgr5 in WT organoids but displayed a less pronounced effect on these genes in LIF KO organoids. SC79 greatly increased the mRNA levels of these genes in LIF KO organoids but displayed a less pronounced effect on them in WT organoids (Fig. 4i). Employing organoids formed by WT or LIF KO; Lgr5-GFP mouse crypts, we found that Wortmanin and Capivasertib greatly decreased stem cell number in WT organoids and exhibited a much less pronounced inhibitory effect on LIF KO organoids, and SC79 greatly increased stem cell number in LIF KO organoids but showed a less pronounced effect on WT organoids (Supplementary Fig. S8d). We further examined whether blocking GSK3β improves the growth of LIF KO organoids. CHIR99021, a specific GSK3β inhibitor^30^, greatly improved the growth, cell proliferation and stem cell number of LIF KO organoids but showed a much less pronounced effect on WT organoids (Fig. 4j and Supplementary Fig. S8e–g).

In addition to AKT, LIF activates JAK/STAT3 and MAPK pathways to mediate some of LIF’s functions. Blocking STAT3 or MAPK pathway by Stattic and SB242235, inhibitors for STAT3 and MAPK, respectively^31,32^, did not have a significant effect on the growth and proliferation of WT or LIF KO organoids (Supplementary Fig. S9). Combined treatment of Wortmannin and Stattic did not show a stronger inhibitory effect on the growth and proliferation of WT or LIF KO organoids than Wortmannin treatment alone (Supplementary Fig. S9). These results suggest that JAK/STAT3 and MAPK pathways are not major mediators for LIF’s function of maintaining ISC function.

β-catenin is upregulated by Wnt ligands in the small intestine^11^. Wnt 3 is a major Wnt ligand in small intestine^14^. In addition, EGF and Dll1 are key ligands that provide support to Lgr5^+^ ISCs^14^. LIF deficiency did not significantly affect the mRNA levels of Wnt 3, EGF and Dll1 in the small intestine (Supplementary Fig. S10).

Collectively, these results suggest that LIF upregulates the β-catenin signaling mainly through the AKT signaling to maintain ISC function.

### LIF deficiency impairs intestinal epithelial regeneration and reduces lifespan after radiation in mice

ISCs are critical for intestinal regeneration after injury, such as IR-induced epithelial renewal. In response to high doses of IR, the intestinal epithelium of mice goes through an apoptotic phase in the first 2 days, which is followed by a proliferative phase showing regeneration of crypts^8^. Due to the interruption of the extremely rapid cell turnover and loss of sufficient crypts in the intestine, proper intestinal mucosal barrier can not be maintained, which is susceptible to infection. Eventually the intestine becomes ulcerated, a phenomena called GI syndrome, leading to death of mice within days^33^.

**Fig. 5:**
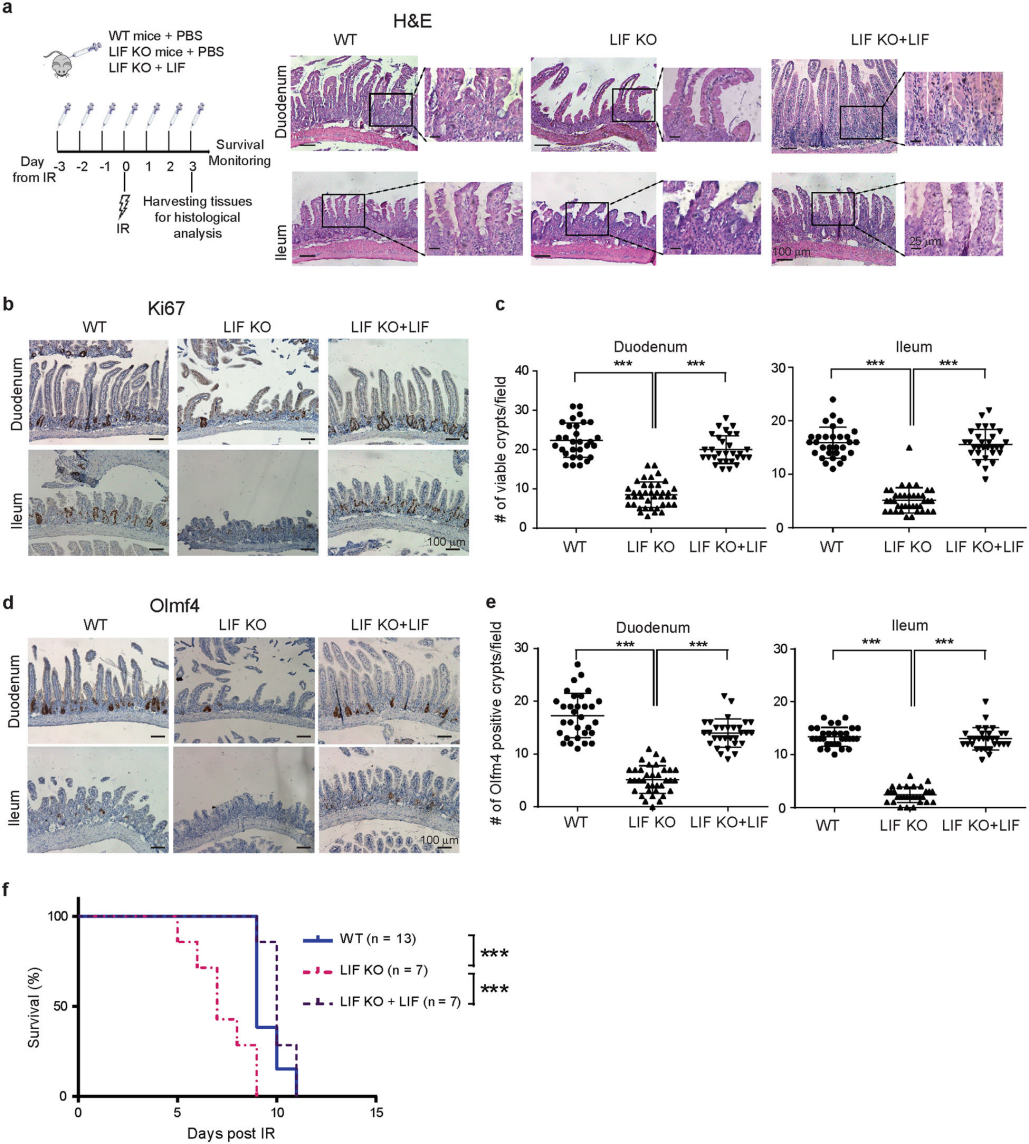
LIF deficiency impairs regeneration of the intestinal epithelium after IR which can be rescued by administering mouse rLIF. **a** The morphology of the duodenum and ileum of WT, LIF KO, and LIF KO mice injected with mouse rLIF (LIF KO + LIF) examined by H&E staining at 72 h after 12 Gy whole-body IR. Mice were irradiated with 12 Gy whole-body IR on day 0, and injected with rLIF (i.p.; 30 ng/g body weight) or vehicle (PBS) twice a day from day −3 to day 3. Left panel: a schematic diagram of experimental procedures; right panels: representative H&E images. **b**, **c** A significantly decreased number of proliferating crypts in LIF KO mice at 72 h post IR, which was rescued by LIF injection. **b** Representative images of IHC staining of Ki67 in the duodenum and ileum of mice post IR. **c** Quantification of viable crypts/field in the duodenum and ileum of mice post IR. **d**, **e** A significantly reduced number of ISCs in LIF KO mice at 72 h post IR, which was rescued by LIF injection. **d** Representative images of IHC staining of Olfm4 in the duodenum and ileum of mice post IR. **e** Quantification of Olfm4 positive crypts/field in the duodenum and ileum of mice post IR. **f** Kaplan–Meier survival curve of WT, LIF KO, and LIF KO + LIF mice post 12 Gy IR. In **c**–**e**, data are presented as mean ± SD. *n* = 30 fields from at least 3 mice/group. ***p < 0.001; Student’s *t*-test. In **f**, ***p < 0.001; Kaplan–Meier survival analysis.

We investigated LIF’s role in IR-induced intestinal epithelial regeneration in mice. The impairment of ISC function in LIF KO mice was exacerbated by 12 Gy whole-body IR. At 72 h after IR, compared with the small intestine of WT mice, the small intestine of LIF KO mice showed more severe epithelial injury, including moderate to severe villous blunting, less lamina propria, disorganized villous architecture, and immature intestinal epithelium (Fig. 5a). In WT mice, numerous enlarged/hyperplastic crypts indicative of regeneration were observed at 72 h after IR (Fig. 5a). In contrast, regeneration was impaired with a dramatic reduction of regenerating crypts in LIF KO mice at 72 h after IR (Fig. 5a–c). Histological analysis revealed that the length and the density of intestinal villi in LIF KO mice were significantly reduced compared with WT mice at 72 h after IR (Fig. 5a and Supplementary Fig. S11). Compared with WT mice, LIF KO mice contained significantly fewer viable crypts (defined as a crypt-like structure containing at least five adjacent Ki67^+^ cells) (Fig. 5b, c), and significantly lower levels of Olfm4 (Fig. 5d, e) in the small intestine. Notably, the impairment of intestinal regeneration in LIF KO mice was largely rescued by administering mice with mouse rLIF (i.p. 30 ng/g body weight, twice/day for 7 days, starting from 3 days before IR) (Fig. 5a and Supplementary Fig. S11). Importantly, administering mice with rLIF increased the number of viable crypts and Olfm4 levels in the small intestine of LIF KO mice to a similar extent as observed in WT mice (Fig. 5b–e).

Consistent with previous reports^34,35^, WT mice subjected to 12 Gy whole-body IR had a median lifespan of 9 days due to the GI syndrome (Fig. 5f). Compared with WT mice, LIF KO mice subjected to 12 Gy IR had a significantly reduced lifespan with a median lifespan of 7 days (*p* < 0.001) (Fig. 5f). This reduction of lifespan can be restored by administering rLIF to LIF KO mice (Fig. 5f). These results indicate that LIF is important for efficient regeneration of the intestinal epithelium, as well as survival of mice upon injury challenge.

### Administering rLIF promotes the regeneration of intestinal epithelium and prolongs lifespan after radiation in WT mice

**Fig. 6:**
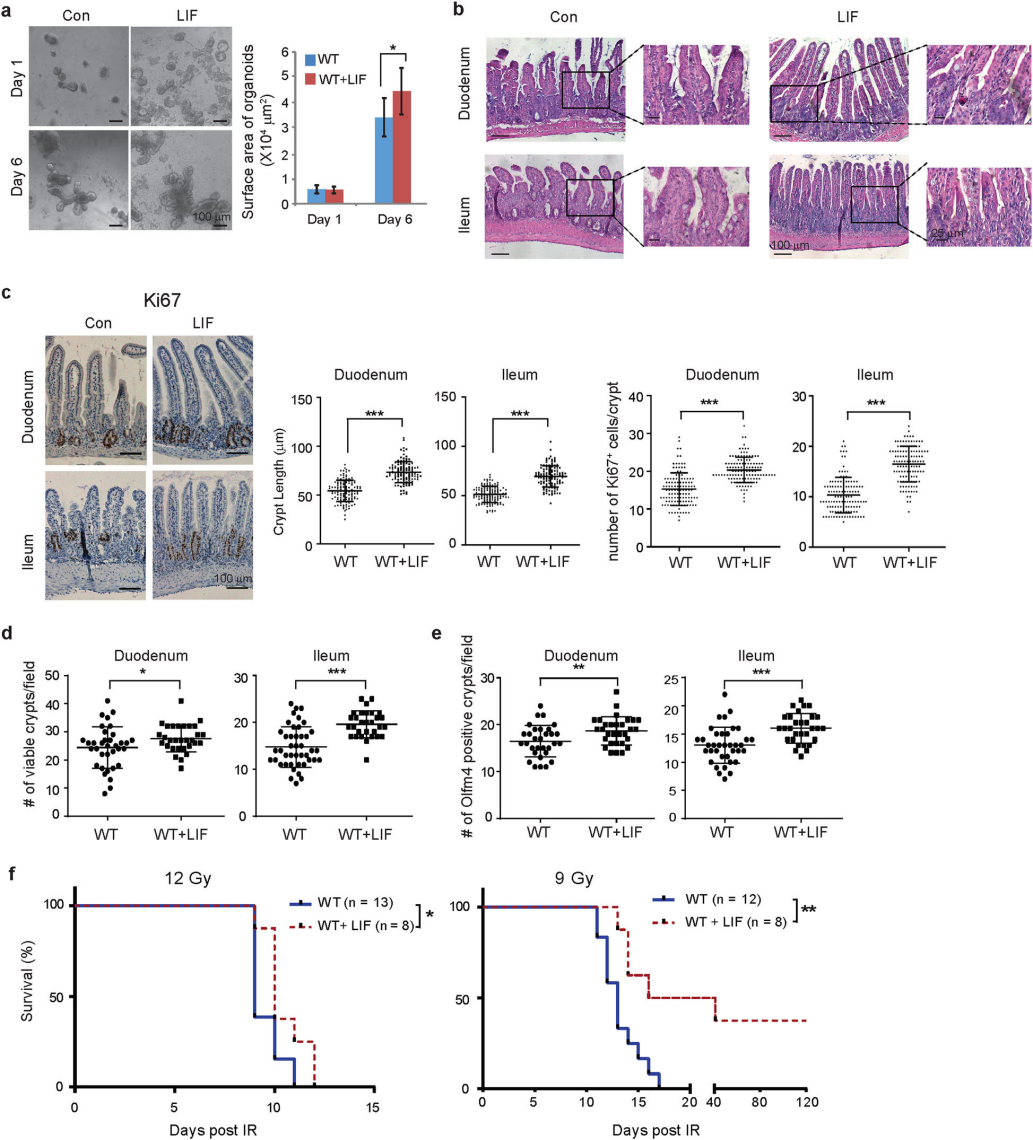
Administering mouse rLIF promotes regeneration of the intestinal epithelium and prolongs survival of WT mice after IR. **a** Supplementing the medium with mouse rLIF (50 ng/mL) promoted the growth of organoids from WT mice. Left panel: representative images of intestinal organoids. Right panel: quantification of organoid surface area. **b** The morphology of the duodenum and ileum of WT mice with or without injection of rLIF (WT + LIF) examined by H&E staining at 72 h after 12 Gy IR. **c** IHC staining of Ki67 in the duodenum and ileum of WT and WT + LIF mice at 72 h after 12 Gy IR. Left panel: representative images of Ki67 IHC of the duodenum and ileum. Middle panel, quantification of crypt length. Right panel: the number of Ki67^+^ cells/crypt. Data are presented as mean ± SD. *n* = 120 crypts from at least 3 mice/group. **d**, **e** Quantification of the number of viable crypts/field (**d**) and Olfm4 positive crypts/field (**e**) in the duodenum and ileum of WT and WT + LIF mice at 72 h post 12 Gy IR. **f** Kaplan–Meier survival curve of WT and WT + LIF mice post 12 Gy whole-body IR (left) and 9 Gy whole-body IR (right). In **a**, **d**, **e**, data are presented as mean ± SD. *n* = 30 fields from at least 3 mice/group. *p < 0.05; **p < 0.01; ***p < 0.001; Student’s *t*-test. In **f**, *p < 0.05; **p < 0.01; Kaplan–Meier survival analysis.

We next investigated whether supplementing LIF can improve adult ISC function and protect against radiation-induced GI syndrome in WT mice. LIF supplementation in culture media promoted the growth of organoids with enlarged surface area (Fig. 6a), suggesting that LIF enhances the clonogenic activity of WT ISCs. To examine whether supplementing LIF has a radioprotective effect on WT mice through promoting the regeneration of intestinal epithelium, mouse rLIF was administered to WT mice *via* i.p. at a dose of 30 ng/g body weight, twice/day for 7 days, starting from 3 days before IR. At 72 h after 12 Gy IR, WT mice with LIF treatment showed much greater intestinal regeneration compared with mice treated with vehicle. LIF treatment increased the length and density of villi and the number of viable crypts in the intestinal tissues of WT mice (Fig. 6b). Compared with control mice, the average length of crypts was much longer, and the average number of proliferating cells which are Ki67 positive in each crypt was significantly higher in WT mice administrated with LIF (Fig. 6c), indicating that LIF enhanced ISC regeneration function. The numbers of viable crypts and Olfm4 levels were also increased in WT mice with LIF treatment, compared with the control mice (Fig. 6d, e). Furthermore, LIF administration significantly prolonged the lifespan of WT mice in response to 12 Gy whole-body IR; LIF-treated mice had a median survival of 10 days whereas control mice had a median survival of 9 days (*p* = 0.03) (Fig. 6f). The protective effect was more obvious when WT mice were subject to 9 Gy whole-body irradiation; while the control group had a median lifespan of 13 days, LIF-treated group had a median lifespan of 28.5 days with ~50% of the group bypassed GI syndrome-induced lethality (*p* = 0.005) (Fig. 6f). These results demonstrate a radioprotective role of LIF in WT mice.

## Discussion

As a highly pleiotropic cytokine, LIF has multiple important functions. The functions of LIF are highly context dependent. For example, LIF was originally identified as an inducer to differentiate myeloid leukemia cells and prevent the proliferation of leukemia cells. Later, LIF was found to maintain the self-renewal function of embryonic stem cells and inhibit their differentiation (for review, see refs. ^1,2^).

Whereas it is well-established that LIF plays a crucial role in maintaining the pluripotency of embryonic stem cells and induced pluripotent stem cells in vitro^1,36^, the role of LIF in adult ISCs in vivo is not well-understood. This study demonstrates an essential role of LIF in regulating adult ISC functions and the intestinal maintenance. In mice, the expression of LIF is observed in several types of cells in the intestinal crypt throughout the intestine in mice. The expression of LIF is also detected in the crypt in human colon tissues, suggesting that LIF has a conserved function in regulating ISCs in mice and humans.

This study shows that the expression of LIF in the stem cell niche enables ISCs and progenitors to proliferate and maintain homeostasis of the intestinal epithelial tissue. LIF deficiency leads to a decrease of the stem cell compartment in the intestine. Compared with WT mice, intestinal crypts of LIF KO mice are smaller and contain less ISCs, Paneth cells and proliferating TA cells. LIF is not only essential to maintain homeostasis of the intestinal epithelium, but also important for the regeneration of epithelium in response to injury. The intestinal epithelial regeneration induced by IR is severely compromised in LIF KO mice. LIF KO mice have a reduced lifespan after 12 Gy whole body IR compared with WT mice. Administering mouse rLIF can rescue the impaired intestinal epithelial regeneration in response to IR and restore the lifespan of LIF KO mice to a similar extent as WT mice. More importantly, LIF promotes the intestinal epithelial regeneration of WT mice after IR and prolongs their lifespans. Thus, the prominent effect of LIF on the stem cell compartment in the intestine highlights a critical role of LIF in regulating the proliferation and self-renewal function of adult stem cells. Further, our results suggest that LIF has the potential to be developed for medical countermeasures against radiation.

This study demonstrates that LIF regulates ISCs’ function by upregulating β-catenin activity mainly through the AKT signaling. β-catenin is a critical regulator of adult stem cells, including ISCs. β-catenin fulfills its stem cell-associated functions through its nuclear translocation to induce its target genes^11,12^. Loss of LIF significantly reduces nuclear localization of β-catenin and decreases the expression of β-catenin target genes. Further, we found that LIF up-regulates β-catenin function through the AKT/GSK3β signaling. AKT activates β-catenin through inactivation of GSK3β, a negative regulator of β-catenin. LIF up-regulates AKT activity in the intestine; loss of LIF decreases AKT activity in the intestine in the LIF KO mice, which in turn increases GSK3β activity, leading to the decreased β-catenin activity. LIF can activate JAK/STAT3 signaling, which plays a central role in maintaining self-renewal and pluripotency of mouse embryonic stem cells^5,36^. LIF can also activate MAPK signaling to mediate some of its functions. However, neither JAK/STAT3 nor MAPK pathway plays a major role in maintaining ISC functions. These observations indicate that the pleiotropic functions of LIF are mediated by different downstream pathways in a highly cell- and tissue-specific manner.

A recent study reported that LIF plays a protective role in mouse experimental colitis models^37^. Under the colitis condition, microbiota dysregulation induces LIF secretion by IECs. LIF protects colitis through regulating the function of lamina propria lymphocytes *via* the STAT4 signaling and repair function of IECs *via* the STAT3/YAP signaling. Results from this study and our study revealed important functions of LIF in the gut under different conditions, including physiological conditions, injury induced by IR, and colitis conditions, through different mechanisms.

To develop LIF as a therapeutic agent to protect or treat radiation-induced GI damage, it is important to ensure the safety of the administration of LIF in humans. Human rLIF has been used in clinical studies for preventing chemotherapy-induced peripheral neurophathy in cancer patients and improving implantation and pregnancy in women with recurrent unexplained implantation failure, and showed a good safety in both studies^38,39^. Nonetheless, the safety of human rLIF at the dosage to be used to protect or treat GI damage needs to be carefully tested.

Taken together, this study reveals a crucial role of LIF in supporting ISC function, promoting regeneration of intestinal epithelium in response to radiation, and protecting against radiation-induced GI syndrome.

## Supplementary information

Supplementary Information

Supplementary Information figure1

Supplementary Information figure2

Supplementary Information figure3

Supplementary Information figure4

Supplementary Information figure5

Supplementary Information figure6

Supplementary Information figure7

Supplementary Information figure8

Supplementary Information figure9

Supplementary Information figure10

Supplementary Information figure11
