# Supplementary material for: LIF is essential for ISC function and protects against radiation-induced gastrointestinal syndrome
Source: Cell Death Dis. 2020 Jul 27;11(7):588. doi: 10.1038/s41419-020-02790-6 (PMC7385639; doi:10.1038/s41419-020-02790-6)
Supplement: Supplementary file 1 — Supplementary Information [file 41419_2020_2790_MOESM1_ESM.docx]

# Supplementary Materials

**Supplementary Table 1. The sequences of the primers for real-time PCR assays**

| Gene | Primer |
| --- | --- |
| *Olfm4* | Forward: 5’ GCCACTTTCCAATTTCAC 3’  Reverse: 5’ GAGCCTCTTCTCATACAC 3’ |
| *Lysozyme* | Forward: 5’ GGTGGTGAGAGATCCCCAAG 3’  Reverse: 5’ CAGACTCCGCAGTTCCGAAT 3’ |
| *Axin 2* | Forward: 5’ TGAGATCCACGGAAACAGC 3’  Reverse: 5’ GTGGCTGGTGCAAAGACAT 3’ |
| *Ascl 2* | Forward: 5’ TCCAGTTGGTTAGGGGGCTA 3’  Reverse: 5’ GCATAGGCCCAGGTTTCTTG 3’ |
| *Lgr5* | Forward: 5’ CCTACTCGAAGACTTACCCAGT 3’  Reverse: 5’ GCATTGGGGTGAATGATAGCA 3’ |
| *Wnt3* | Forward: 5’ CTTCTAATGGAGCCCCACCT 3’  Reverse: 5’ GAGGCCAGAGATGTGTACTGC 3’ |
| *EGF* | Forward: 5’ AGCAGCCCCTTCCCTAAGA 3’  Reverse: 5’ AGTGTGTCCGTCCTCCGAA 3’ |
| *Dll4* | Forward: 5’ TTCCAGGCAACCTTCTCCGA 3’  Reverse: 5’ ACTGCCGCTATTCTTGTCCC 3’ |
| *β-Actin* | Forward: 5’ GAACCCTAAGGCCAACCGTGAAAAGATGAC 3’  Reverse: 5’ GCAGGATGGCGTGAGGGAGAGCA 3’ |

**Supplementary Figures**

**Supplementary Figure 1. The expression of LIF in the intestinal epithelium of WT mice at embryonic and postnatal development stages.** IF staining of LIF and Olfm4 in the intestinal epithelium of E14.5, E18.5 embryos and P7, P15 pups. H&E staining of an E14.5 embryo was also shown.

**Supplementary Figure 2. Genotype of embryos and offspring from breeding pairs of mice heterozygous for LIF (a) and body weight of 8-week-old WT and LIF KO mice (b).** For b, data are presented as mean ± SD. **: *p*<0.01; Student’s *t*-test.

**Supplementary Figure 3. Differentiated cell types, including goblet cells and tuft cells, in the intestinal villi, and the morphology of colon of WT and LIF KO mice. a.** Goblet cells in the intestinal villi of WT and LIF KO mice were visualized by staining with Alcian blue (left panels). Right panel: quantification of the number of goblet cells in the intestinal villi (n=120 villi from at least 3 mice/group) of WT and LIF KO mice. Data are presented as mean ± SD. *p*=0.16; Student’s *t*-test. **b.** IHC staining of DCLK, a marker of tuft cells. The black arrows point to tuft cells with positive staining of DCLK. **c.** LIF deficiency impairs the development of the colon in mice. Left panels: representative H&E staining images of the colon of WT and LIF KO mice. Right panel: quantification of the crypt depth (n= 300 crypts from at least 5 mice/group) in the colon of WT and LIF KO mice. ***: *p*<0.001; Student’s *t*-test.

**Supplementary Figure 4. The apoptosis of the intestinal epithelium examined by IHC staining of cleaved caspase-3 in the small intestine of WT and LIF KO mice.** Representative images of IHC staining of the cleaved caspase-3 in the duodenum and ileum of WT and LIF KO mice.

**Supplementary Figure 5. The number of ISCs reduced in LIF KO mice compared with WT mice. a.** The percentage of Lgr5-GFP+ cells is greatly decreased in colonic epithelium of LIF KO; Lgr5-GFP mice. Left panels: representative images of flow cytometry analysis of Lgr5- GFP+ cells in colonic epithelium of WT; Lgr5-GFP and LIF KO; Lgr5-GFP mice. Right panel: quantification of the percentage of Lgr5-GFP+ cells in colonic epithelium. n=3 mice/group. *: *p*<0.05; Student’s *t*-test. **b.** IHC staining of CD44 in the duodenum and ileum of WT and LIF KO mice.

**Supplementary Figure 6. LIF deficiency reduces the number of Paneth cells in the small intestine and intestinal organoids. a-c.** LIF deficiency reduces the number of Paneth cells per crypt and the production of lysozyme in the small intestine. **a.** IF staining of lysozyme in the duodenum and ileum of WT and LIF KO mice. **b.** Quantification of the number of Paneth cells per crypt in the small intestine of WT and LIF KO mice. n=20 crypts/group. **c.** Quantification of

the relative fluorescence intensity per cell with positive lysozyme staining. n !8 sections from at least 3 mice/group. **d.** Representative images showing IF staining of UEA-1, a Paneth cell marker, in WT, LIF KO and LIF KO organoids supplemented with LIF. For **b** & **c**, data are presented as mean " SD., **: *p*<0.01; Student’s *t*-test.

**Supplementary Figure 7. Administering recombinant mouse LIF rescues the impaired intestinal homeostasis of LIF KO mice.** LIF KO mice were administered with recombinant mouse LIF for constitutively 7 days. WT and LIF KO mice administered with PBS were served as the control. The intestinal tissues were harvested on day 8. **a-c.** Quantification of the villus length (n=120 villi from at least 3 mice/group) (**a**), villus density (n=48 fields from at least 3 mice/group) (**b**), and crypt depth (n=120 crypts from at least 3 mice/group) (**c**) in the small intestine of WT, LIF KO, and LIF KO with LIF injection mice. The villus length and crypt depth were normalized to the average weight of WT mice with the same age and gender. **d-f.**

Quantification of the number (**d**) and percentage (**e**) of Ki67 positive cells per crypt, and the number of Olfm4 positive cells per crypt (**f**). n=120 crypts from at least 3 mice/group. Data are presented as mean ± SD. **: *p*<0.01; ***: *p*<0.001; One-way ANOVA followed by SNK test.

**Supplementary Figure 8. LIF upregulates the β-catenin signaling *via* AKT in the small intestine. a** & **b.** Quantification of the percentage of intestinal organoids formation in WT and LIF KO intestinal organoids with or without treatments of Wortmannin or Capivasertib (**a**) or SC79 (**b**). **c.** IF staining of Ki67 in WT and LIF KO intestinal organoids with or without LIF or SC79 treatment at day 6. **d.** IF staining of GFP in the intestinal organoids from WT; Lgr5-GFP and LIF KO; Lgr5-GFP mice with or without treatments of Wortmannin, Capivasertib, LIF or SC79. **e.** Quantification of the percentage of intestinal organoids formation in WT and LIF KO intestinal organoids with or without CHIR99021 treatment. **f.** IF staining of Ki67 in WT and LIF KO intestinal organoids with or without CHIR99021 treatment at day 6. **g.** IF staining of GFP in the intestinal organoids from WT; Lgr5-GFP and LIF KO; Lgr5-GFP mice with or without CHIR99021 treatment. For **a**, **b** & **e**, n ≥100/group, *: *p*<0.05; **: *p*<0.01; ***: *p*<0.001; Fisher’s exact test.

# Supplementary Figure 9. Blocking STAT3 or MAP signaling pathway does not have a significant effect on the growth and proliferation of WT or LIF KO organoids.

Quantifications of the surface area (**a**) and the percentage of intestinal organoids formation (**b**) of WT and LIF KO intestinal organoids with or without treatments of Wortmannin (1 μM), Stattic (STAT3 inhibitor, 2 μM), or SB242235 (MAPK inhibitor, 1 μM). In **a**, data are presented as mean ± SD. n ≥ 30/group, *: *p*<0.05; ***: *p*<0.001; One-way ANOVA followed by SNK test. In **b**, n ≥100/group, **: *p*<0.01; ***: *p*<0.001; Fisher’s exact test.

**Supplementary Figure 10. Relative mRNA expression levels of Wnt 3, EGF, and DLL1 in the small intestine of WT and LIF KO mice.** mRNA levels were determined by quantitative realtime PCR and normalized with β-actin. Data are presented as mean ± SD. n = 6 mice/group; *p* value was calculated by Student’s *t*-test.

# Supplementary Figure 11. Administering recombinant mouse LIF rescues the impaired regeneration of intestinal epithelium in LIF KO mice after IR.

Quantification of the villus length (n=120 villi from at least 3 mice/group) (left panels) and villus density (n=48 fields from at least 3 mice/group) (right panels) in the small intestine of naïve WT, or WT, LIF KO, and LIF KO with LIF injection mice after IR. The villus length was normalized to the average weight of naïve WT mice with the same age and gender. Data are presented as mean ± SD. ***: *p*<0.001; One-way ANOVA followed by SNK test.
