# Supplementary figures and images for: LIF is essential for ISC function and protects against radiation-induced gastrointestinal syndrome
Source: Cell Death Dis. 2020 Jul 27;11(7):588. doi: 10.1038/s41419-020-02790-6 (PMC7385639; doi:10.1038/s41419-020-02790-6)

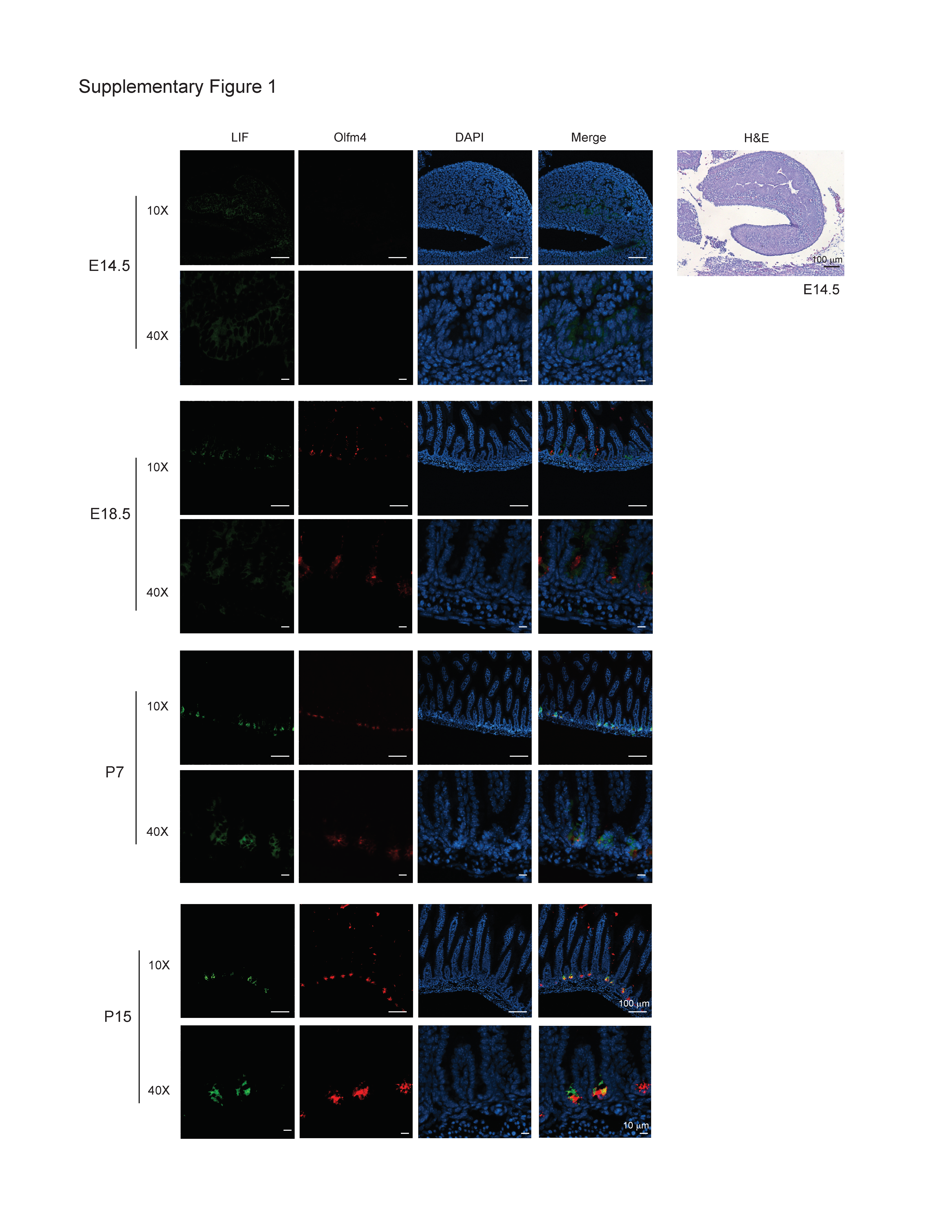

Supplement: Supplementary file 2 — Supplementary Information figure1 [file 41419_2020_2790_MOESM2_ESM.png]

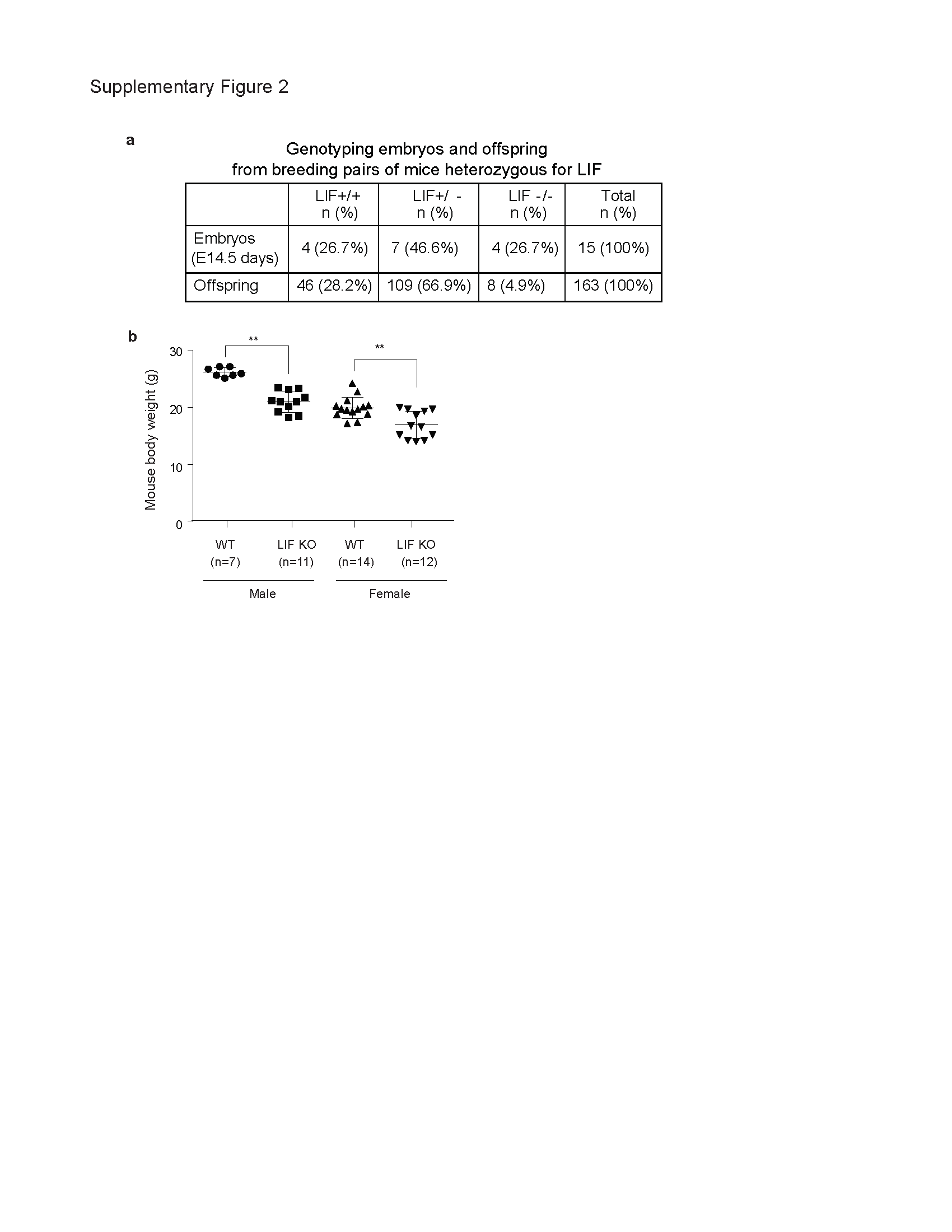

Supplement: Supplementary file 3 — Supplementary Information figure2 [file 41419_2020_2790_MOESM3_ESM.png]

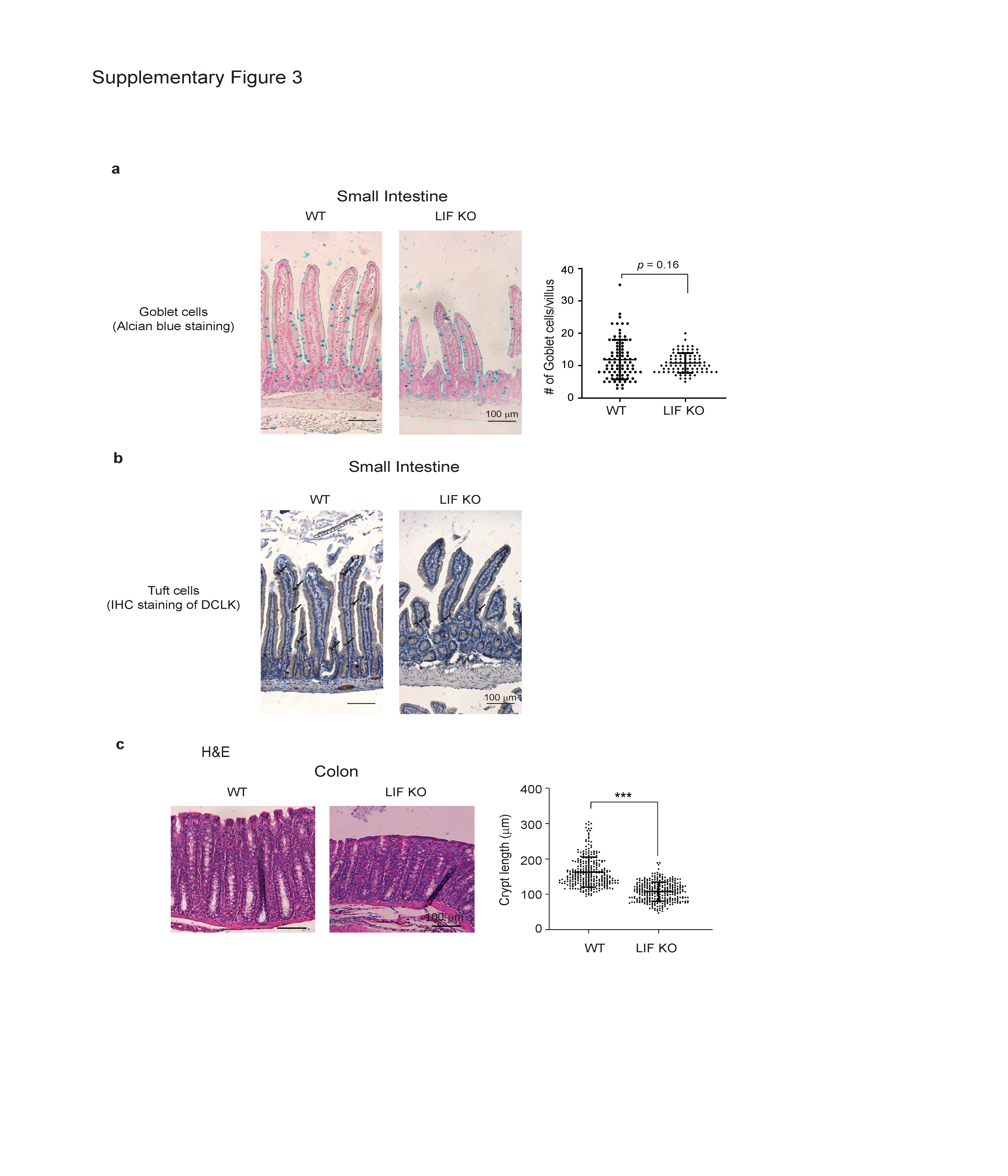

Supplement: Supplementary file 4 — Supplementary Information figure3 [file 41419_2020_2790_MOESM4_ESM.png]

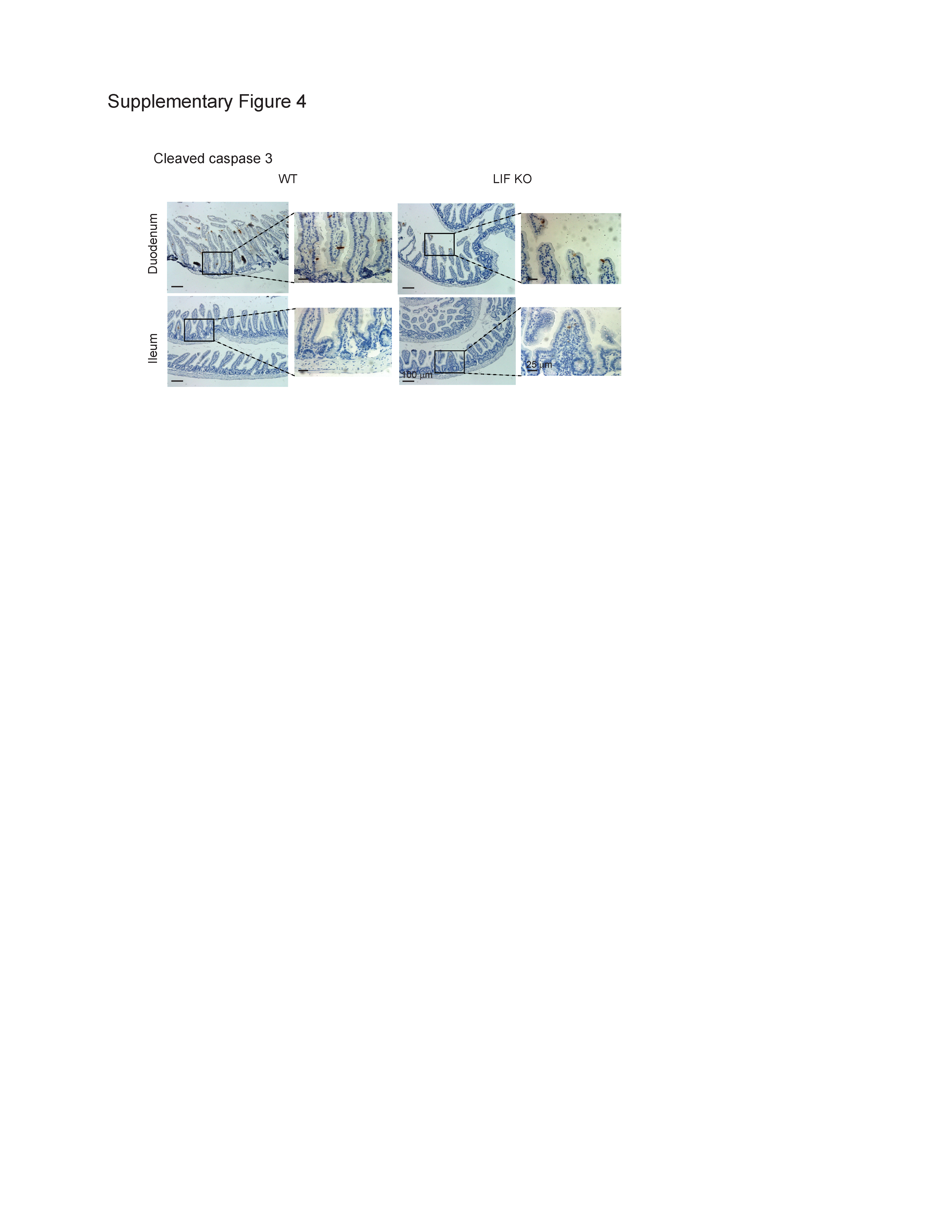

Supplement: Supplementary file 5 — Supplementary Information figure4 [file 41419_2020_2790_MOESM5_ESM.png]

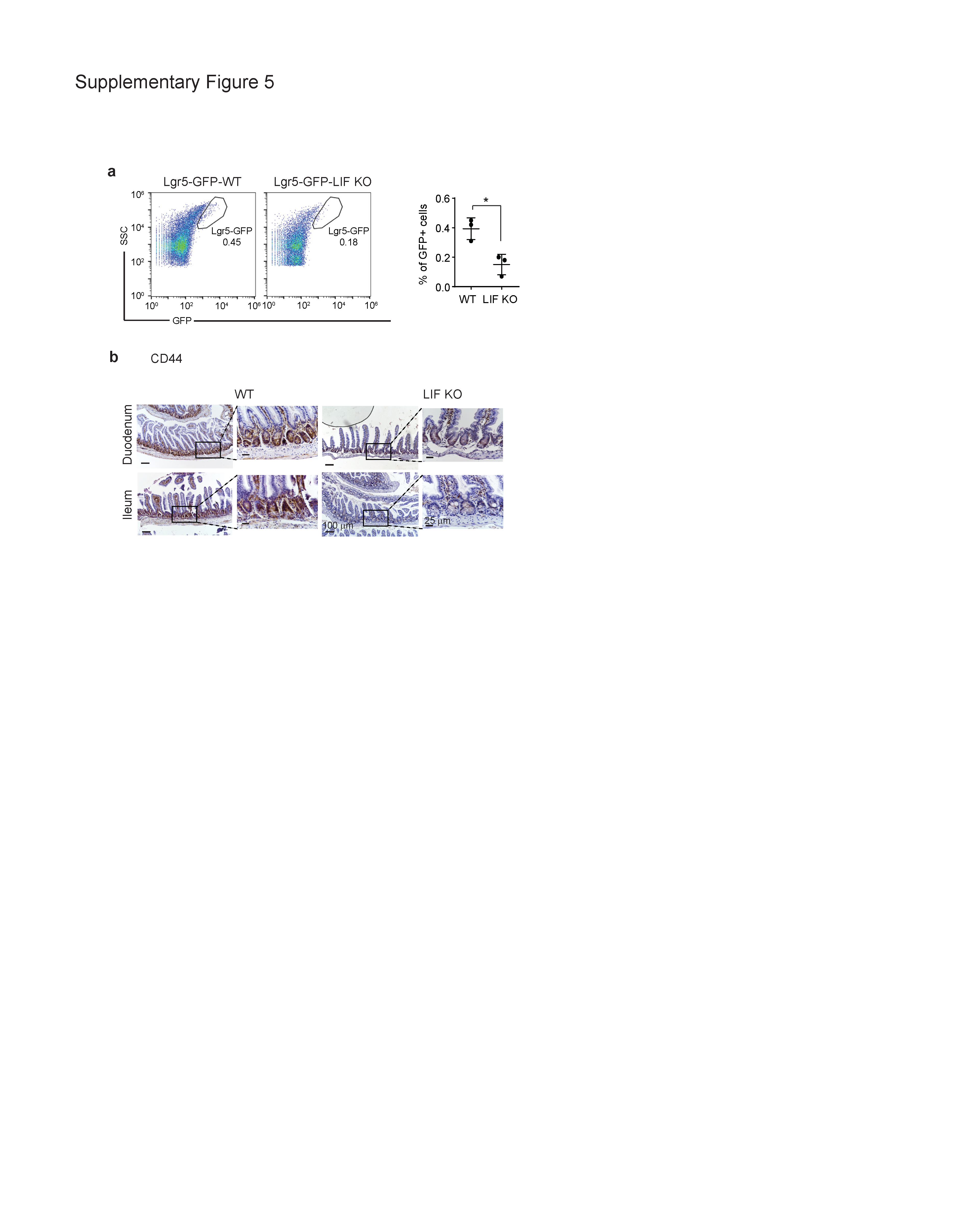

Supplement: Supplementary file 6 — Supplementary Information figure5 [file 41419_2020_2790_MOESM6_ESM.png]

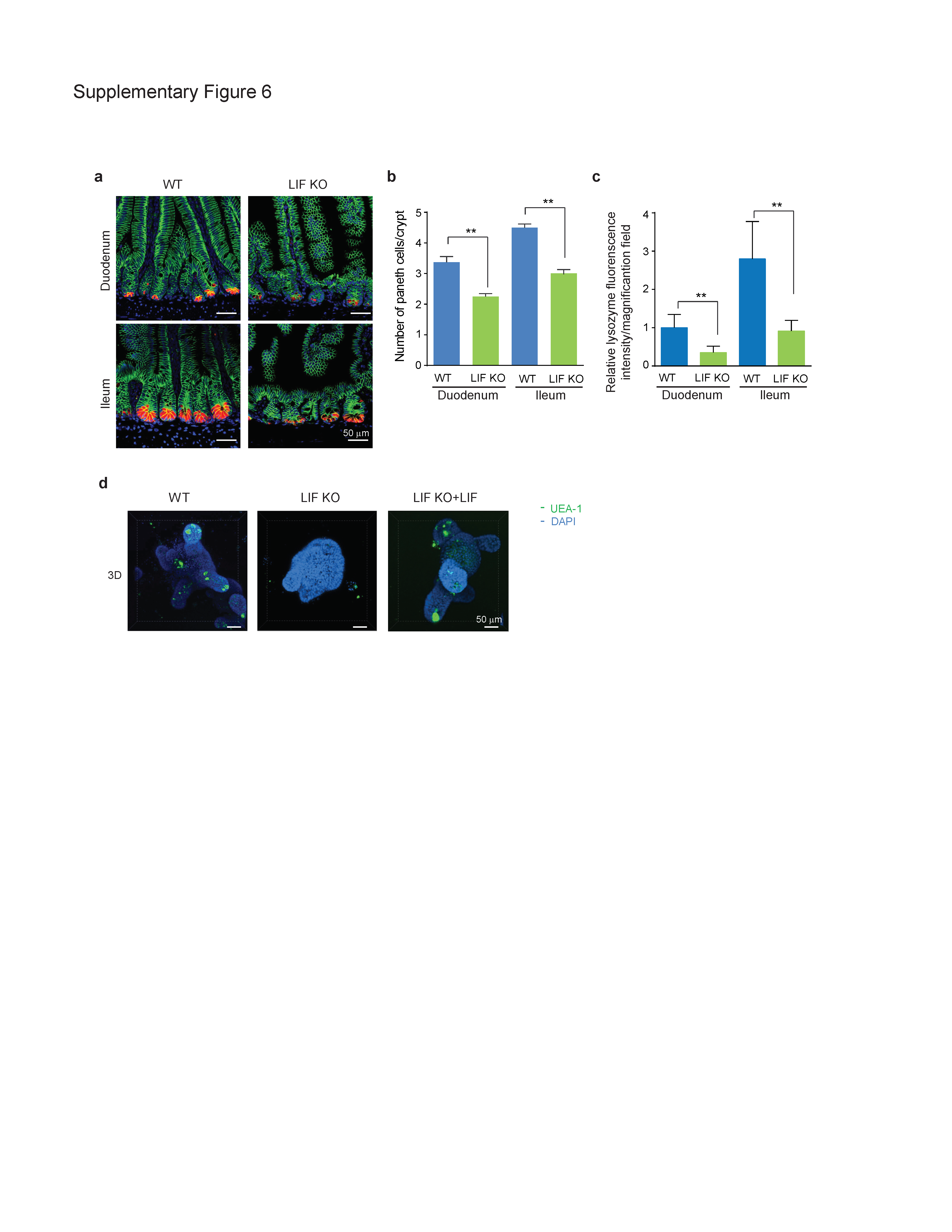

Supplement: Supplementary file 7 — Supplementary Information figure6 [file 41419_2020_2790_MOESM7_ESM.png]

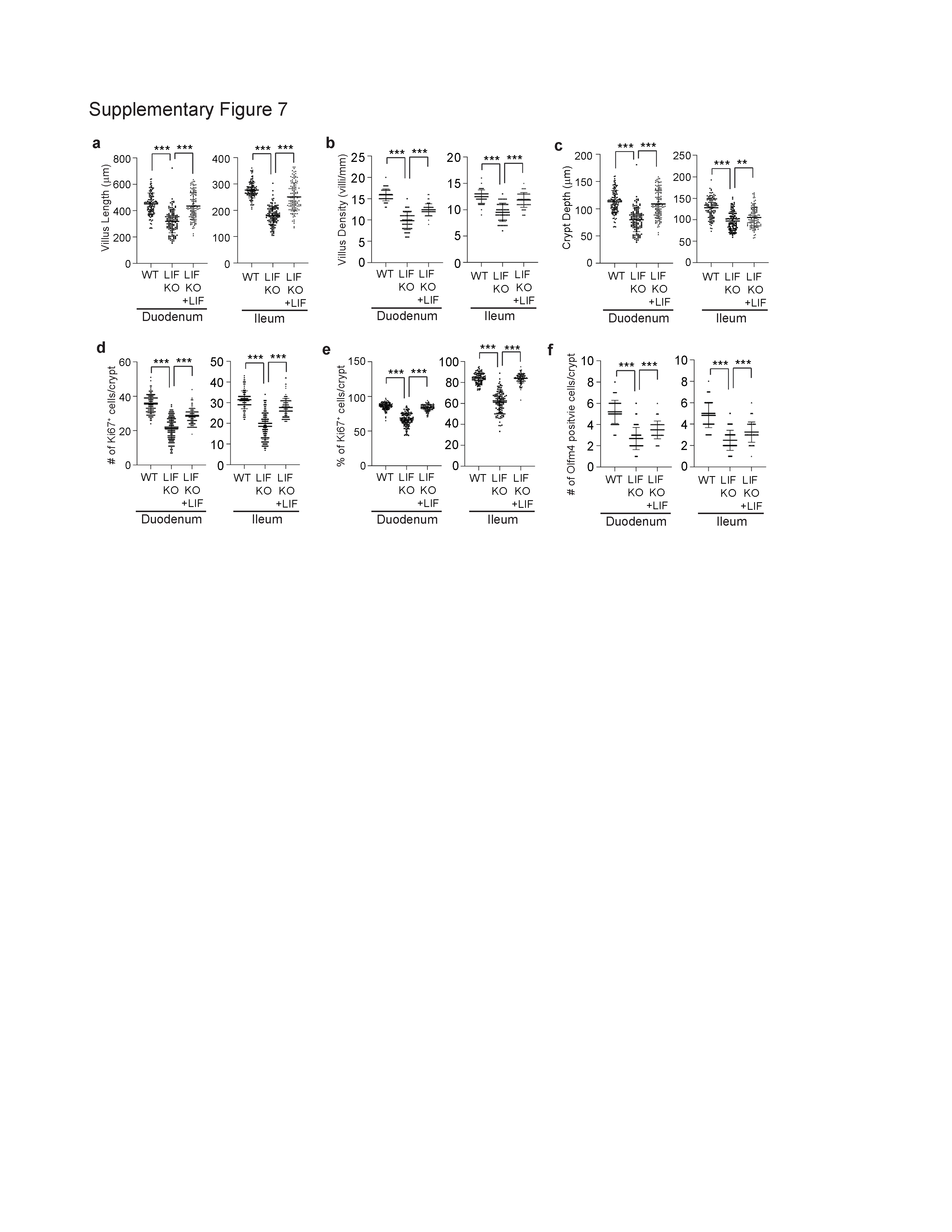

Supplement: Supplementary file 8 — Supplementary Information figure7 [file 41419_2020_2790_MOESM8_ESM.png]

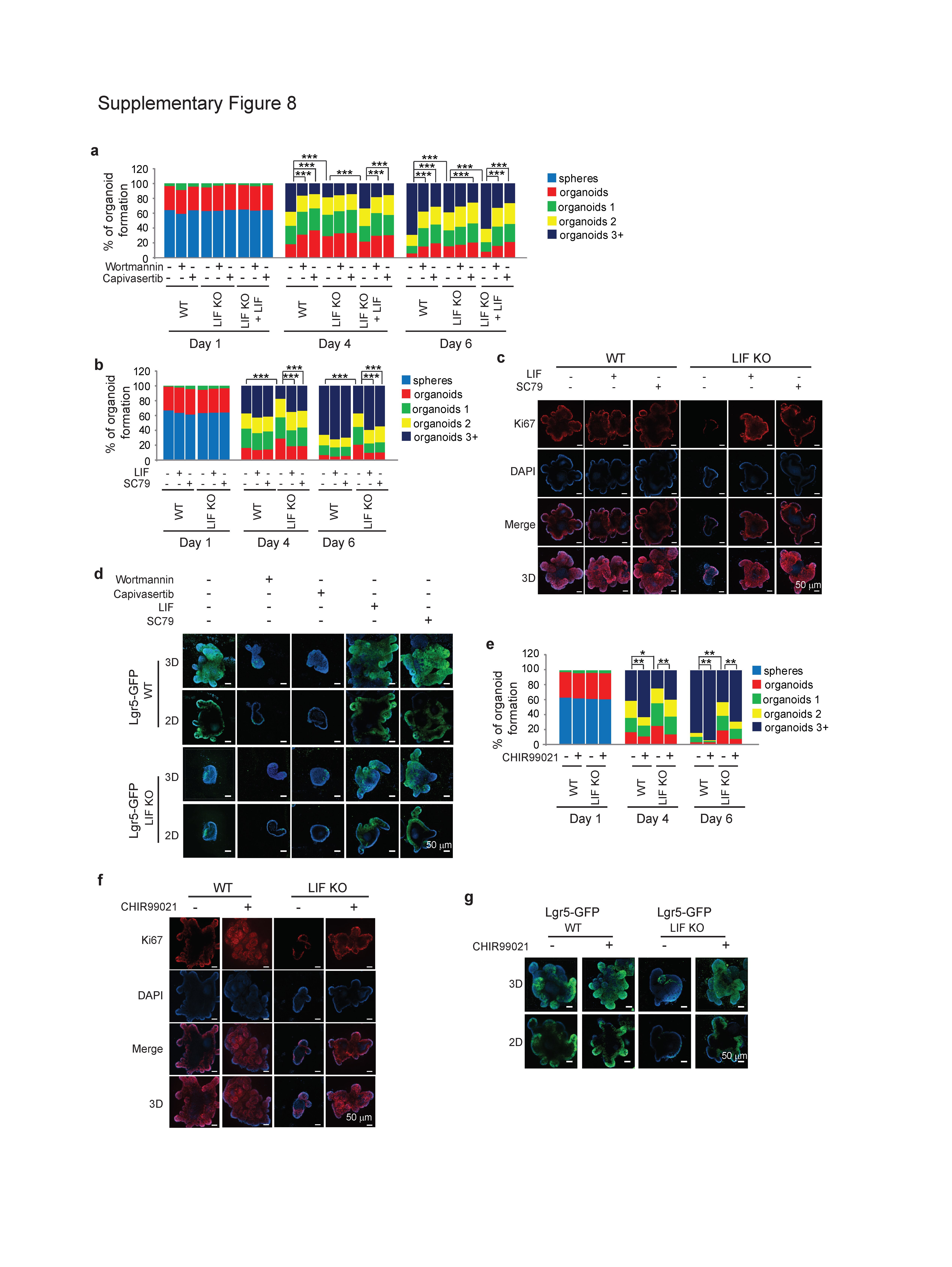

Supplement: Supplementary file 9 — Supplementary Information figure8 [file 41419_2020_2790_MOESM9_ESM.png]

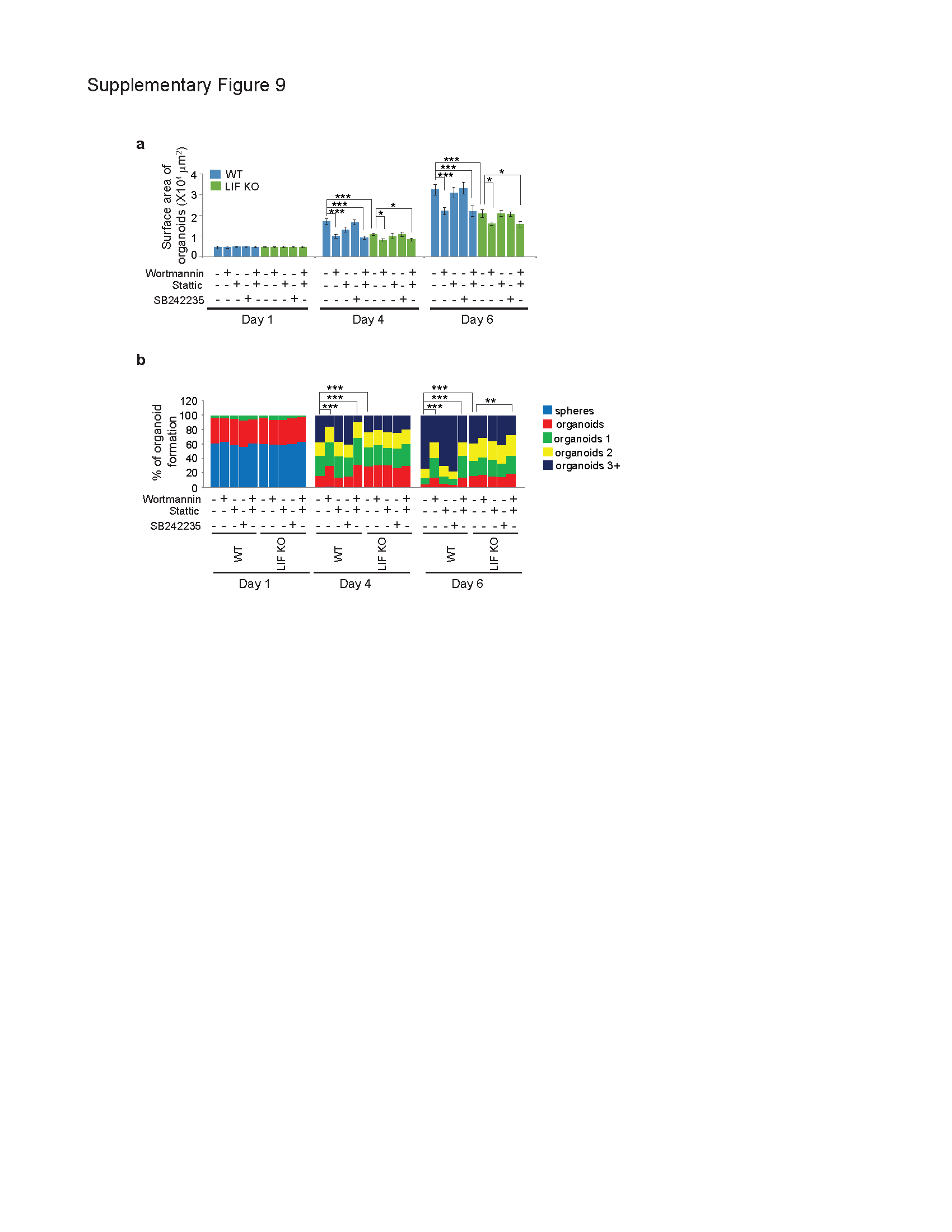

Supplement: Supplementary file 10 — Supplementary Information figure9 [file 41419_2020_2790_MOESM10_ESM.png]

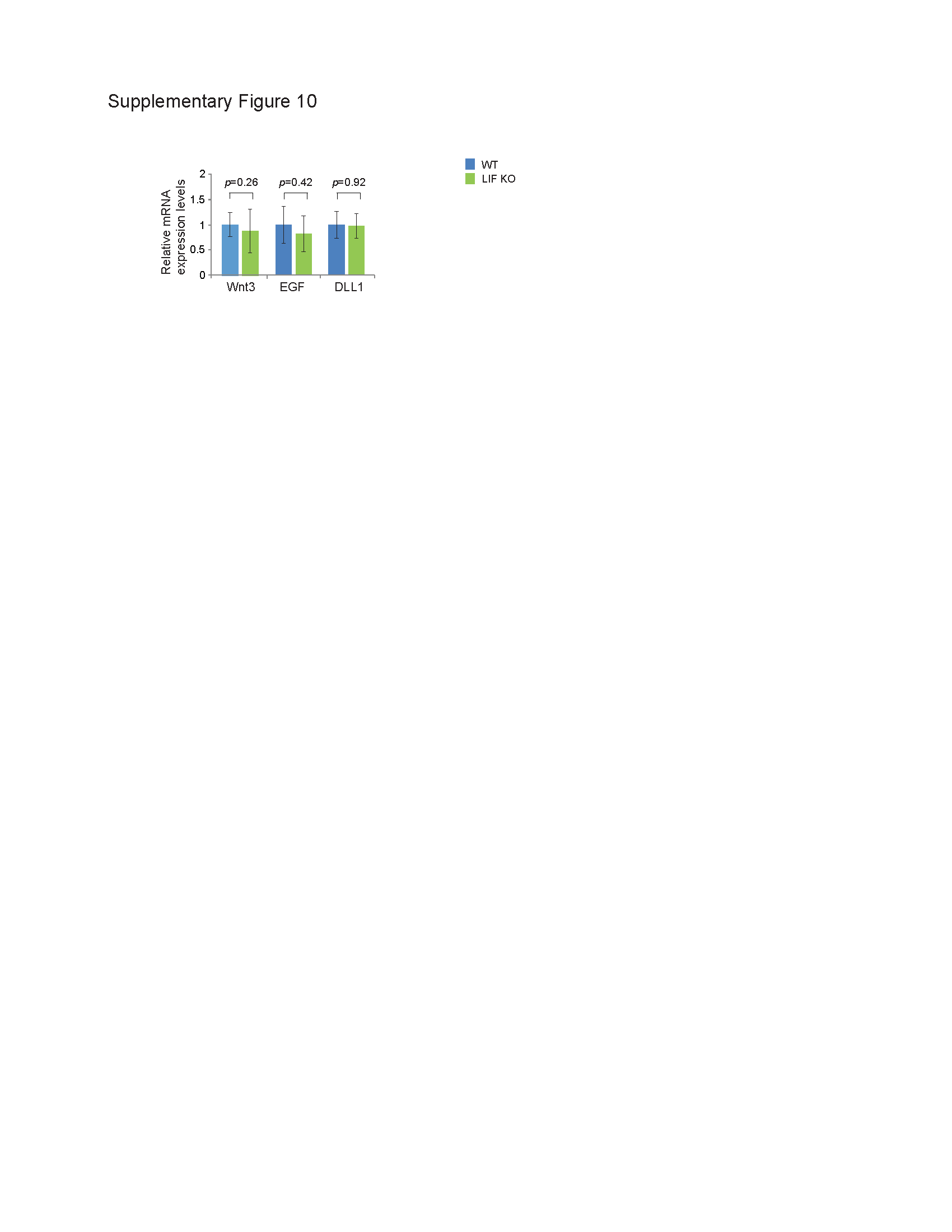

Supplement: Supplementary file 11 — Supplementary Information figure10 [file 41419_2020_2790_MOESM11_ESM.png]

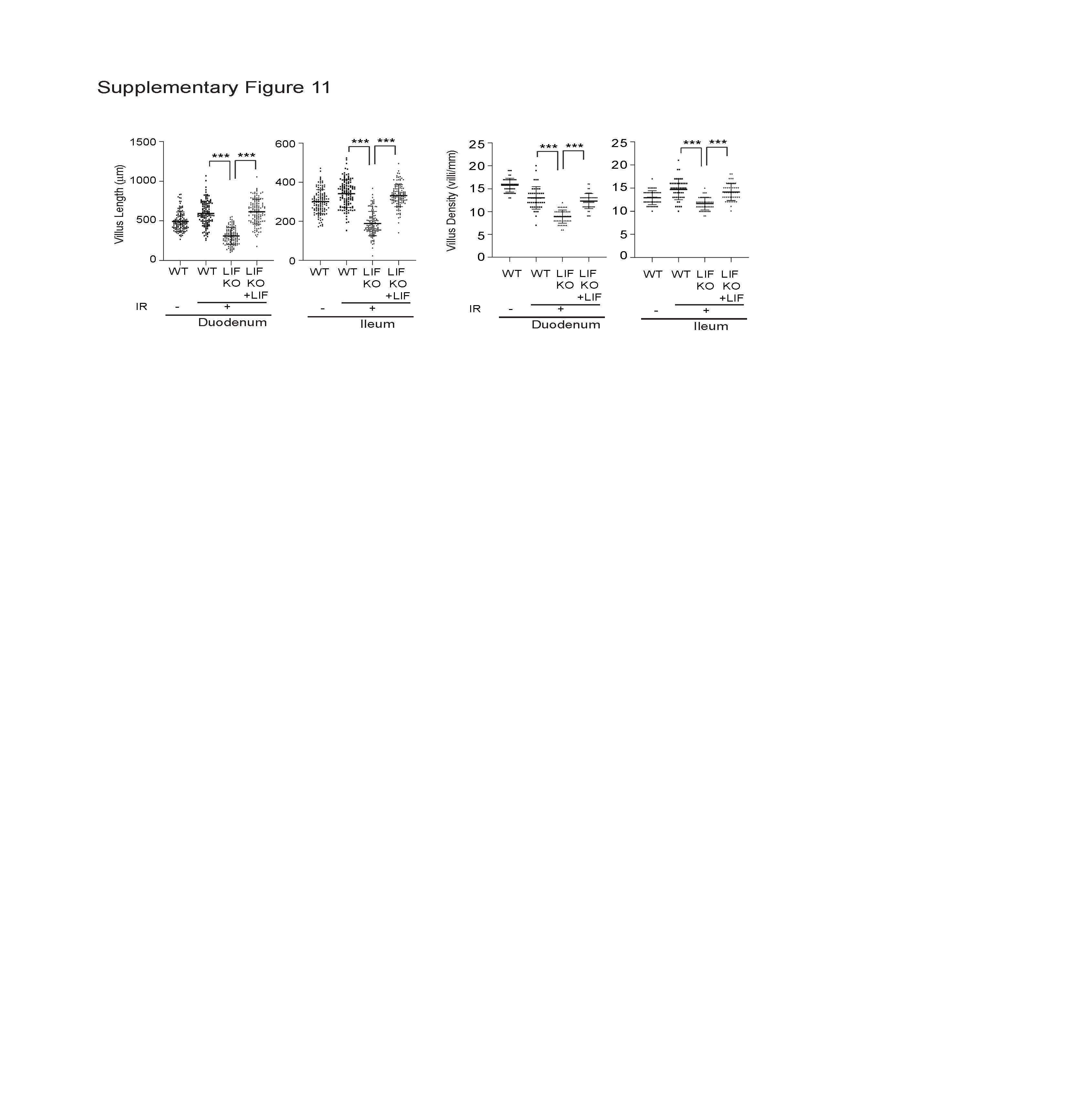

Supplement: Supplementary file 12 — Supplementary Information figure11 [file 41419_2020_2790_MOESM12_ESM.png]
